# DER containing two consecutive GTP-binding domains plays an essential role in chloroplast ribosomal RNA processing and ribosome biogenesis in higher plants

**DOI:** 10.1093/jxb/ert360

**Published:** 2013-11-23

**Authors:** Young Jeon, Chang Sook Ahn, Hyun Ju Jung, Hunseung Kang, Guen Tae Park, Yeonhee Choi, Jihwan Hwang, Hyun-Sook Pai

**Affiliations:** ^1^Department of Systems Biology, Yonsei University, Seoul 120-749, Korea; ^2^Department of Plant Biotechnology, College of Agriculture and Life Sciences, Chonnam National University, Gwangju 500-757, Korea; ^3^School of Biological Sciences, Seoul National University, Seoul 151-;747, Korea; ^4^Department of Microbiology, Pusan National University, Busan 609-735, Korea

**Keywords:** Chloroplast abnormality, *Nicotiana benthamiana*, ribosomal RNA processing, ribosome association, RNA binding, virus-induced gene silencing.

## Abstract

This study investigated protein characteristics and physiological functions of DER (Double Era-like GTPase) of higher plants. *Nicotiana benthamiana* DER (NbDER) contained two tandemly repeated GTP-binding domains (GD) and a C-terminal domain (CTD) that was similar to the K-homology domain involved in RNA binding. Both GDs possessed GTPase activity and contributed to the maximum GTPase activity of NbDER. NbDER fused to green fluorescent protein was localized primarily to chloroplast nucleoids. *Arabidopsis der* null mutants exhibited an embryonic lethal phenotype, indicating an essential function of DER during plant embryogenesis. Virus-induced gene silencing of NbDER resulted in a leaf-yellowing phenotype caused by disrupted chloroplast biogenesis. NbDER was associated primarily with the chloroplast 50S ribosomal subunit *in vivo*, and both the CTD and the two GD contributed to the association. Recombinant proteins of NbDER and its CTD could bind to 23S and 16S ribosomal RNAs *in vitro*. Depletion of NbDER impaired processing of plastid-encoded ribosomal RNAs, resulting in accumulation of the precursor rRNAs in the chloroplasts. NbDER-deficient chloroplasts contained significantly reduced levels of mature 23S and 16S rRNAs and diverse mRNAs in the polysomal fractions, suggesting decreased translation in chloroplasts. These results suggest that DER is involved in chloroplast rRNA processing and ribosome biogenesis in higher plants.

## Abstract (precis)

Chloroplast-localized DER (*D*ouble *Er*a-like GTPase) contains two consecutive GTP-binding domains, each of which possesses GTPase activity. DER binds to 23S and 16S ribosomal RNAs, and plays an essential role in chloroplast ribosomal RNA processing and ribosome biogenesis in higher plants

## Introduction

GTPases are widely distributed among prokaryotes, archaea, and eukaryotes. They play critical roles in protein translation, protein translocation, and signal transduction (Caldon and March, 2003; Verstraeten *et al.*, 2011). GTPases generally cycle between a GDP-bound inactive state and a GTP-bound active state, and the accompanying conformational changes are crucial for their function as molecular switches in diverse cellular processes. GTPases contain conserved sequence motifs called the G motifs (G1–G5), which are crucially involved in guanine nucleotide binding, catalysis, and effector binding.

The universally conserved bacterial GTPases play important roles in ribosome function, including ribosome biogenesis and protein translation (Caldon and March, 2003; Verstraeten *et al.*, 2011). However, except for a few GTPases that belong to translation machinery, the regulatory mechanisms for most GTPases that function as ribosome assembly factors are still poorly understood. Among these bacterial GTPases, Era has been implicated in 30S ribosome assembly, cell cycle regulation, and energy metabolism (Ahnn *et al.*, 1986; Minkovsky *et al.*, 2002; Verstraeten *et al.*, 2011). Era binds to 16S ribosomal RNA and 30S ribosome subunits *in vitro*; recently, the ribosome-binding mechanism of Era with its GTP-binding domain and K-homology domain has been characterized (Chen *et al.*, 1999; Sharma *et al.*, 2005). Obg co-sediments with 30S and 50S ribosome subunits and interacts with 16S and 23S rRNAs and with specific ribosomal proteins in a GTP-dependent fashion (Sato *et al.*, 2005). Obg plays a role in ribosome assembly and has additional functions in stress response, sporulation, and DNA replication (Jiang *et al.*, 2006; Verstraeten *et al.*, 2011). DER (EngA, YphC, or YfgK) is an essential GTPase with a unique structure: it contains two tandemly repeated GTP-binding domains followed by a unique C-terminal domain (Robinson *et al.*, 2002). The GTPase activities of both GTP-binding domains are indispensible for cellular functions of DER in *Escherichia coli* (Bharat *et al.*, 2006). DER interacts with 50S ribosome subunits in a GTP-dependent manner, and DER deficiency results in the accumulation of rRNA precursors and an aberrant ribosome profile (Hwang and Inouye, 2006). DER-deficient *E. coli* cells contain 50S subunits that lack ribosomal proteins L16, L27, and L36, indicating that DER plays a role in biogenesis and stability of the 50S subunit (Hwang and Inouye, 2006). DER has also been proposed to have a role in chromosome segregation, stringent response, and cell-wall assembly (Hwang and Inouye, 2008; Verstraeten *et al.*, 2011).

Homologues of bacterial GTPases are found in plants as organellar GTPases. The Era homologue ERG of *Antirrhinum majus* (snapdragon) is required for embryonic development and, during postembryonic growth, the gene is preferentially expressed in metabolically active cells (Ingram *et al.*, 1998). ERG regulates plant growth, possibly by controlling mitochondrial division. The Obg homologue OBGC of *Arabidopsis thaliana* is localized in the stroma and inner envelope membrane and appears to be associated primarily with ribosome biogenesis processes in chloroplasts (Bang *et al.*, 2012). Rice OsNOA1/RIF1, a homologue of bacterial YqeH GTPase, is targeted to chloroplasts and its RNAi mutant contains a reduced level of plastid-encoded proteins and nuclear-encoded chloroplast protein complexes (Liu *et al.*, 2010). Taken together, these results suggest essential functions in plant organelles for the conserved nuclear-encoded GTPases of prokaryotic origin.

This study determined the *in vivo* functions of DER in higher plants. DER homologues are found only in bacteria and plants, and the plant DER protein is targeted to chloroplasts. Plant DER proteins have two GTP-binding domains in tandem, and both of the domains have GTPase activity. Virus-induced gene silencing (VIGS) of DER in *Nicotiana benthamiana* results in defects in rRNA processing and ribosome biogenesis in chloroplasts. The possible roles of plant DER during chloroplast development are discussed.

## Materials and methods

### Virus-induced gene silencing

Functional genomics using tobacco rattle virus (TRV)-based VIGS was carried out in *N. benthamiana* as described (Cho *et al.*, 2004; Kang *et al.*, 2010; Jeon *et al.*, 2012). Various NbDER cDNA fragments were amplified by PCR and cloned into the pTV00 vector containing part of the TRV genome (Cho *et al.*, 2004) using *Bam*HI and *Apa*I sites. The recombinant pTV00 plasmids and pBINTRA6 vector containing RNA1, which is required for virus replication, were separately transformed into *Agrobacterium tumefaciens* GV3101. The third leaf of *N. benthamiana* (3-week-old plants) was pressure-infiltrated with the *Agrobacterium* suspension. The fourth leaf above the infiltrated leaf was used for real-time quantitative reverse-transcription (RT)-PCR to detect gene silencing.

### *Agrobacterium*-mediated transient expression

Agroinfiltration was carried out as described previously (Voinnet *et al.*, 2003). Agrobacterial cultures (GV3101) containing various constructs fused to the CaMV35S promoter were adjusted to OD_600_ 0.6 in MES buffer (10mM MES, pH 7.5, 10mM MgSO_4_). The suspension was incubated with acetosyringone for 2–3h at a final concentration of 150 μM, and infiltrated into leaves of wild-type *N. benthamiana* plants. In all experiments, *Agrobacterium* C58C1 carrying the 35S:p19 construct (Voinnet *et al.*, 2003) was coinfiltrated to achieve maximum levels of protein expression. Expressed proteins were analysed at 48h post-infiltration.

### Real-time quantitative RT-PCR

Real-time quantitative RT-PCR was carried out as described previously (Jeon *et al.*, 2012). The primer sets used for detection of endogenous DER transcripts are given in Supplementary Table S1 (available at JXB online).

### RNA gel blot analysis

For RNA gel blot analysis, total RNA was prepared with TRIzolTM Reagent (Gibco-BRL) following the manufacturer’s instructions. RNA gel blot analyses were performed with approximately 20 μg total RNA as described previously (Jeon *et al.*, 2012). To generate probes, cDNAs were amplified by PCR using published sequences and cloned for sequence verification. Probes were labelled with a DecaLabel DNA Labeling Kit (Thermo Scientific). The primer sets used for PCR amplification of the probes and the probe sizes are detailed in Supplementary Table S1.

### DAPI staining

DAPI staining and detection by confocal laser scanning microscopy was performed as described in Cho *et al.*, (2004).

### Confocal microscopy for subcellular localization of NbDER

NbDER cDNAs corresponding to the full-length coding region (from Met-1 to Ala-651) and the N-terminal transit peptide (from Met-1 to Ser-60) were cloned into the sGFP plasmid (Cho *et al.*, 2004) to generate NbDER:GFP and TP:GFP fusion proteins, respectively. The GFP fusion constructs, alone or in combination with STF:RFP (Jeon *et al.*, 2012) and SiR:RFP constructs (Kang *et al.*, 2010), were introduced into protoplasts isolated from *N. benthamiana* seedlings as described previously (Cho *et al.*, 2004). After 24h, expression of the GFP and RFP fusion constructs was monitored by confocal laser scanning microscopy (LSM 510, Carl Zeiss).

### Transmission electron microscopy

Cotyledons and leaves were fixed with 2.5% (v/v) glutaraldehyde and with 1% osmium tetraoxide, followed by dehydration through an ethanol series, and embedded in Spurr’s resin (EM Sciences, USA). Thin sections were prepared with a LKB III ultramicrotome and stained sequentially with 5% uranyl acetate and 3% lead citrate, and observed under a JEOL 1200 EXII transmission electron microscope.

### Immunoblotting

Membrane preparation and Western blotting were performed according to the manufacturer’s instructions using polyclonal rabbit antibodies against rbcL, cyt f, D1, and atpB (1:5000, 1:3000, 1:10 000, and 1:5000 dilution, respectively; Agrisera), and horseradish peroxidase-conjugated goat anti-rabbit IgG antibodies (1:5000 dilution; GE Healthcare). Signals were detected using an electrochemiluminescence kit (Amersham Pharmacia) on Kodak X-ray films. Signal intensities were quantified using the Analysis Life Science Research imaging system (Olympus).

### Chloroplast fractionation and NbDER localization

The NbDER:GFP construct was agroinfiltrated into leaves of *N. benthamiana* plants. After 48h, chloroplast stroma and thylakoid membrane fractions were prepared from the infiltrated leaves as described previously (Kwon and Cho, 2008). The supernatant and pellet fractions were analysed by SDS-PAGE and immunoblotting using the polyclonal antibodies against GFP (Clontech; 1:3000), rbcL (Agrisera; 1:5000), and D1 (Agrisera; 1:10 000) as described previously (Jeon *et al.*, 2012).

### Purification of recombinant proteins

To purify recombinant proteins of NbDER and its mutants for GTPase assays, the corresponding NbDER cDNA fragments were PCR-amplified and cloned into the pMAL^TM^c2 or pMAL-c2 vector (New England Biolabs). The MBP fusion proteins were purified using amylose resin following the manufacturer’s instructions (New England Biolabs). Purified proteins of MBP:NbDER, MBP:PM1, MBP:PM2, and MBP:PM1/2 were concentrated using Amicon Ultra Centrifugal Filters (Millipore). To purify MBP:CTD for RNA-binding assays, the NbDER cDNA fragment corresponding to amino acid residues 520–651 was amplified by PCR and cloned into the pMAL^TM^c2 or pMAL-c2 vector (New England Biolabs).

### RNA binding assay

To prepare 16S and 23S rRNA, the cDNAs encoding full-length 16S and 23S rRNA were cloned into the pGEM T-easy vector. The constructs were digested with *Bam*HI restriction enzyme, and RNAs were prepared by *in vitro* transcription using T7 RNA polymerase (Promega). For RNA binding assays, the RNA substrates were incubated with the purified recombinant MBP fusion proteins in binding buffer (10mM Tris-HCl, pH 7.5, 50mM NaCl, 1mM EDTA, 7.4% glycerol) on ice for 30min. The reaction mixtures were loaded on 0.8% agarose gel, and RNA bands were visualized by UV light after ethidium bromide staining or by PhosphorImager (GE Healthcare Life Sciences). For EMSA competition assays, 25 pmol of the recombinant proteins were incubated with variable ratios of radiolabelled (200ng) and unlabeled RNAs, ranging from 1:0 to 1:20. A sequence-nonspecific ^33^P-labelled RNA substrate (~160 nucleotides in length) was prepared by transcribing *Bam*HI-digested pET-22b(+) plasmid using T7 RNA polymerase as described previously (Jeon *et al.*, 2012). For RNA–protein interaction analysis, the labelled sequence-nonspecific RNAs (30ng) were incubated with purified recombinant proteins in binding buffer (10mM Tris-HCl, pH 8.0, 50mM NaCl, 1mM EDTA, and 7% glycerol) for 30min on ice. The mixture was separated on 6% non-denaturing polyacrylamide gel, and RNA bands were detected by PhosphorImager.

### GTPase assay

The turnover rate (*k*_cat_) of recombinant proteins of NbDER and its mutants was measured as described previously (Im *et al.*, 2011). A reaction mixture containing 3 μM recombinant proteins and 1mM GTP in GTPase assay buffer (20mM HEPES pH 8, 1mM MgCl_4_, 0.5mM DTT, and 1mM NaN_3_) was incubated at room temperature for 18h. The released phosphate was quantified using the Biomol green reagent (Biomol Research Laboratories) according to the manufacturer’s protocol. The catalytic constant was derived from the equation *k*_cat_ = *V*_max_/*C*_recombinant protein_.

### Sucrose density gradient analysis

GFP-fusion proteins of NbDER and its variants were expressed in *N. benthamiana* leaves by agroinfiltration, and the leaf extracts were fractionated through 15–55% sucrose density gradients as described previously (Barkan 1993; Williams and Barkan, 2003). Proteins extracted from the fractions were separated by SDS-PAGE and subjected to immunoblotting with anti-GFP antibodies (Clontech) and anti-RPL10 antibodies (Santa Cruz Biotechnology).

For polysome analysis, leaf extracts of TRV and TRV:NbDER VIGS plants were fractionated through 15–55% sucrose density gradients. Total RNA was extracted from sucrose density gradient fractions and subjected to RNA gel blot analysis.

## Results

### Identification of plant DER

This study carried out functional genomics to identify genes that regulate chloroplast development using TRV-based VIGS in *N. benthamiana* (Cho *et al.*, 2004; Kang *et al.*, 2010; Jeon *et al.*, 2012). This screening revealed that gene silencing of *N. benthamiana* DER encoding a 651-amino-acid polypeptide caused severe leaf yellowing. The ChloroP algorithm (http://www.cbs.dtu.dk/services/ChloroP/, last accessed 25 October 2013) identified a chloroplast transit peptide of 60 amino acids at the N-terminus of *N. benthamiana* DER (NbDER; Genbank accession number KC846070; Supplementary Fig. S1A). NbDER is predicted to contain two consecutive GTP-binding domains (GD1 and GD2) and a C-terminal domain (CTD) that is similar to the K-homology domain. The amino acid sequence of NbDER was aligned with its homologous sequences from *Arabidopsis* (AtDER; At3g12080), *Zea mays* (ZmDER; ACL53683), *E. coli* (EcDER; AAC75564), *Bacillus subtilis* (YphC; AAC83966), and *Thermotoga maritima* (TmDER; AAD36514) (Supplementary Fig. S1B). Compared with plant DER, prokaryotic DER sequences lacked the transit peptide and the N-terminal region enriched with acidic amino acids. The strongest sequence conservation between plant and prokaryotic DER was found in the two GDs; the CTD also contained well-conserved residues (Supplementary Fig. S1B). The sequence alignment revealed well-conserved amino acid residues called the G motifs (G1–G5) in two GDs of plant and prokaryotic DER proteins (Supplementary Fig. S2).

The tertiary structure of NbDER and *Arabidopsis* DER was predicted using an automated homology modelling server [(PS)^2^: Protein Structure Prediction Server; http://ps2.life.nctu.edu.tw/, last accessed 25 October 2013] with the GDP-bound form of *B. subtilis* DER (YphC) as template (Supplementary Fig. S3). The computational model reveals that the mature protein of *N. benthamiana* and *Arabidopsis* DER is very similar to YphC in the overall structure, except the N-terminal region that is not present in YphC. The X-ray crystal structure of *B. subtilis* YphC and *T. maritima* DER reveals that the two GDs do not directly interact with each other, but pack at either side of the CTD (Robinson *et al.*, 2002; Muench *et al.*, 2006), which is also observed in plant DER proteins (Supplementary Fig. S3). The CTD of the plant and prokaryotic DER is highly basic in amino acid composition and, despite the limited sequence homology, it shows a similar topology to that of the K-homology domain that binds to RNA (Grishin, 2001). It has been proposed that GTP binding to YphC triggers a conformational change that stimulates the interaction between the K-homology-like domain and RNA (Muench *et al.*, 2006). The predicted conserved structure of the plant and prokaryotic DER may suggest a conserved function of these proteins.

### Embryonic lethality of *Arabidopsis* T-DNA knockout mutants of DER

Knockout mutations affecting chloroplast proteins of diverse functions caused embryonic lethality (Tzafrir *et al.*, 2004). *DER* is a single-copy gene in *Arabidopsis* genome. According to the SeedGenes Project database (http://www.seedgenes.org/, last accessed 25 October 2013), the *Arabidopsis der* mutants carrying a T-DNA insertion in the upstream region of the gene or in the sixth exon exhibited an embryonic lethal phenotype, designated as EMB2738. Using differential interference contrast microscopy, this study observed phenotypes during embryogenesis for three different alleles of *der* mutation with T-DNA inserted in the first exon (Supplementary Fig. S4A). The three *der* insertion mutants all exhibited embryonic lethality, with the abnormal embryos developmentally arrested at the heart stage (Supplementary Fig. S4B) or the globular stage (Supplementary Fig. S4C). All of these mutants exhibited a mixture of the two embryo-defective phenotypes in a single silique. These results confirm that DER is essential for plant embryo development.

### Chloroplast localization of NbDER

**Fig. 1.**
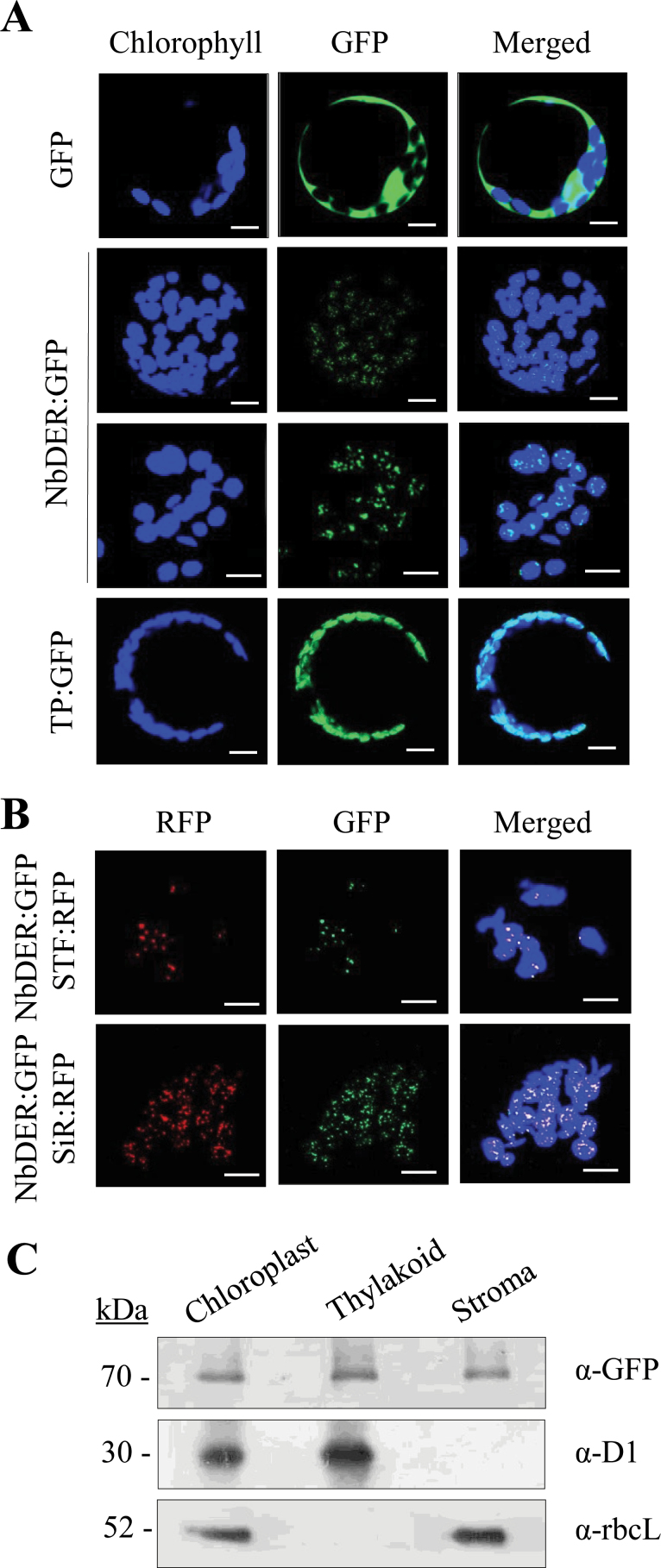
Chloroplast targeting of NbDER. (A) *N. benthamiana* protoplasts were transformed with GFP fusion constructs of full-length NbDER (NbDER:GFP) and its N-terminal transit peptide (TP:GFP); localization of the fluorescent signals was examined by confocal laser scanning microscopy. Chloroplasts were visualized by chlorophyll autofluorescence (pseudo-coloured blue); bars = 10 μm. (B) *N. benthamiana* protoplasts were co-transformed with NbDER:GFP and STF:RFP or with NbDER:GFP and SiR:RFP fusion constructs before observation; both SiR and STF were enriched in chloroplast nucleoids; bars = 5 μm. (C) Localization of NbDER in the stroma and thylakoid fractions of chloroplasts; intact chloroplasts, stroma, and thylakoid membranes were fractionated from *N. benthamiana* leaves agroinfiltrated with the NbDER:GFP fusion construct for expression, and subjected to Western blotting using anti-GFP antibody; D1 and rbcL are markers for thylakoid membranes and stroma, respectively.

To investigate the subcellular localization of DER in plants, this work generated fusion proteins in which full-length NbDER protein (Met-1 to Ala-651) or its putative transit peptide (TP) (Met-1 to Ser-60) was fused to green fluorescent protein (GFP) under the control of the CaMV35S promoter to generate NbDER:GFP and TP:GFP, respectively (Fig. 1A). DNA constructs encoding these GFP fusion proteins were introduced into protoplasts isolated from *N. benthamiana* seedlings, and gene expression was analysed by confocal laser scanning microscopy following the protocol described in Materials and methods. Green fluorescent signals of NbDER:GFP were detected as distinct particles within chloroplasts, whereas the TP:GFP signal was evenly distributed in chloroplasts (Fig. 1A). Next, the protoplasts were co-transformed with DNA constructs encoding the fusion proteins of red fluorescent protein (RFP) with sulphite reductase (SiR:RFP; Sekine *et al.*, 2007; Kang *et al.*, 2010) or RFP with a chloroplast transcription-stimulation factor (STF:RFP; Jeon *et al.*, 2012) (Fig. 1B). Both SiR and STF were associated with chloroplast nucleoids, but also were present in the stroma (Sekine *et al.*, 2007; Kang *et al.*, 2010; Jeon *et al.*, 2012). Both SiR and STF were identified as components of plastid transcriptionally active chromosomes (Pfalz *et al.*, 2006). The NbDER:GFP signal overlapped with the SiR:RFP and STF:RFP signals as distinct particles within chloroplasts, suggesting the association of NbDER with chloroplast nucleoids (Fig. 1B). This result is consistent with a previous report that DER localizes in chloroplast nucleoid-enriched proteomes from maize leaves (Majeran *et al.*, 2012).

### NbDER localization in both stromal and thylakoid membrane fractions

NbDER:GFP was expressed in *N. benthamiana* leaves by agroinfiltration, and chloroplast subfractions were purified from the leaves at 2 d after infiltration. Western blot analysis with anti-GFP antibody revealed that NbDER:GFP protein was localized in both the stromal and thylakoid membrane fractions (Fig. 1C). As controls, rbcL (Rubisco large subunit) was found only in the stromal fraction, whereas the D1 subunit of photosystem II was detected only in the thylakoid membrane fraction (Fig. 1C).

### GTPase activities of NbDER and its variants

**Fig. 2.**
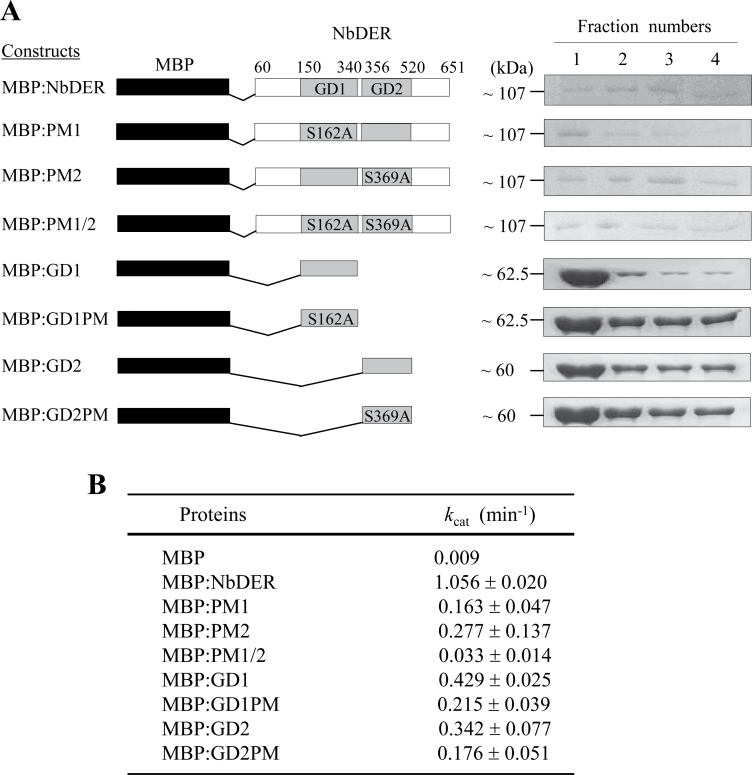
GTPase activity of NbDER and its variants. (A) Schematic drawings of MBP fusion proteins of NbDER and its variants (left panel), and purified recombinant proteins (right panel). Eluted proteins were visualized by Coomassie staining (fractions 1−4). After purification, MBP:NbDER, MBP:PM1, MBP:PM2, and MBP:PM1/2 proteins were further concentrated. (B) GTPase activities of recombinant NbDER and its variants; values are mean ± standard deviation of triplicate experiments.

To investigate the functional importance of each GTP-binding domain (GD) in NbDER, this work determined the GTPase activities of NbDER and its site-specific and deletion mutants (Fig. 2). First, recombinant proteins of NbDER (lacking the N-terminal chloroplast transit peptide), PM1 (S162A mutation), PM2 (S369A mutation), PM1/2 (mutations in both S162A and S369A), GD1 (GD1), GD1PM (S162A mutation in GD1), GD2 (GD2), and GD2PM (S369A mutation in GD2) were prepared as maltose-binding protein (MBP) fusion proteins (Fig. 2A, left panel). The corresponding cDNA fragments were cloned into the pMAL expression vector and expressed in *E. coli*. The recombinant MBP fusion proteins and MBP were affinity-purified using the N-terminal MBP tag, which yielded polypeptides of approximately 60–107kDa (Fig. 2A, right panel). MBP:NbDER, MBP:PM1, MBP:PM2, and MBP:PM1/2 proteins were concentrated after purification. The kinetic constant (*k*_cat_) of MBP:NbDER and other variants (Fig. 2B) were determined. At steady state, the *k*_cat_ of NbDER was 1.056min^–1^, which was comparable to the *k*_cat_ of 1.167min^–1^ for *E. coli* DER and the *k*_cat_ of 0.833min^–1^ for *T. maritima* DER (Robinson *et al.*, 2002; Bharat *et al.*, 2006). The GD1 and GD2 of NbDER showed *k*_cat_ values of 0.429 and 0.342min^–1^, respectively. This result indicated that both GTP-binding domains possessed GTPase activity and that both domains contributed to the maximum activity of the full-length enzyme. MBP alone showed little GTPase activity.

A previous report showed that Ser15 in GD1 and Ser217 in GD2 were crucial residues of the phosphate-binding loop of *E. coli* DER and that expression of the DER variants containing the S16A or S217A mutation could not rescue the lethal phenotype of the *der* deletion mutant of *E. coli* (Bharat *et al.*, 2006). These conserved serine residues were found in the G1 motif of GD1 and GD2 in NbDER (Ser162 and Ser369), which were subsequently mutagenized to alanine (Fig. 2A). The S162A or S369A mutation in NbDER (PM1 or PM2) reduced the *k*_cat_ values to 0.163 and 0.277min^–1^, respectively, and mutation of both residues (PM1/2) almost completely abolished the GTPase activity (*k*_cat_ = 0.033min^–1^). These results suggest that both GTP-binding domains have a significant and cooperative impact on the GTPase activity of NbDER (Fig. 2B). S162A and S369A mutations introduced into the truncated domains GD1 and GD2 (GD1PM and GD2PM) also decreased the GTPase activity of the wild-type GD1 and GD2, but the effect was less significant compared with the effects of the same mutations to the full-length NbDER (Fig. 2B). These data demonstrate that both GD1 and GD2 are crucial for GTPase activity of NbDER.

### VIGS phenotypes and silencing of the endogenous NbDER transcripts

**Fig. 3.**
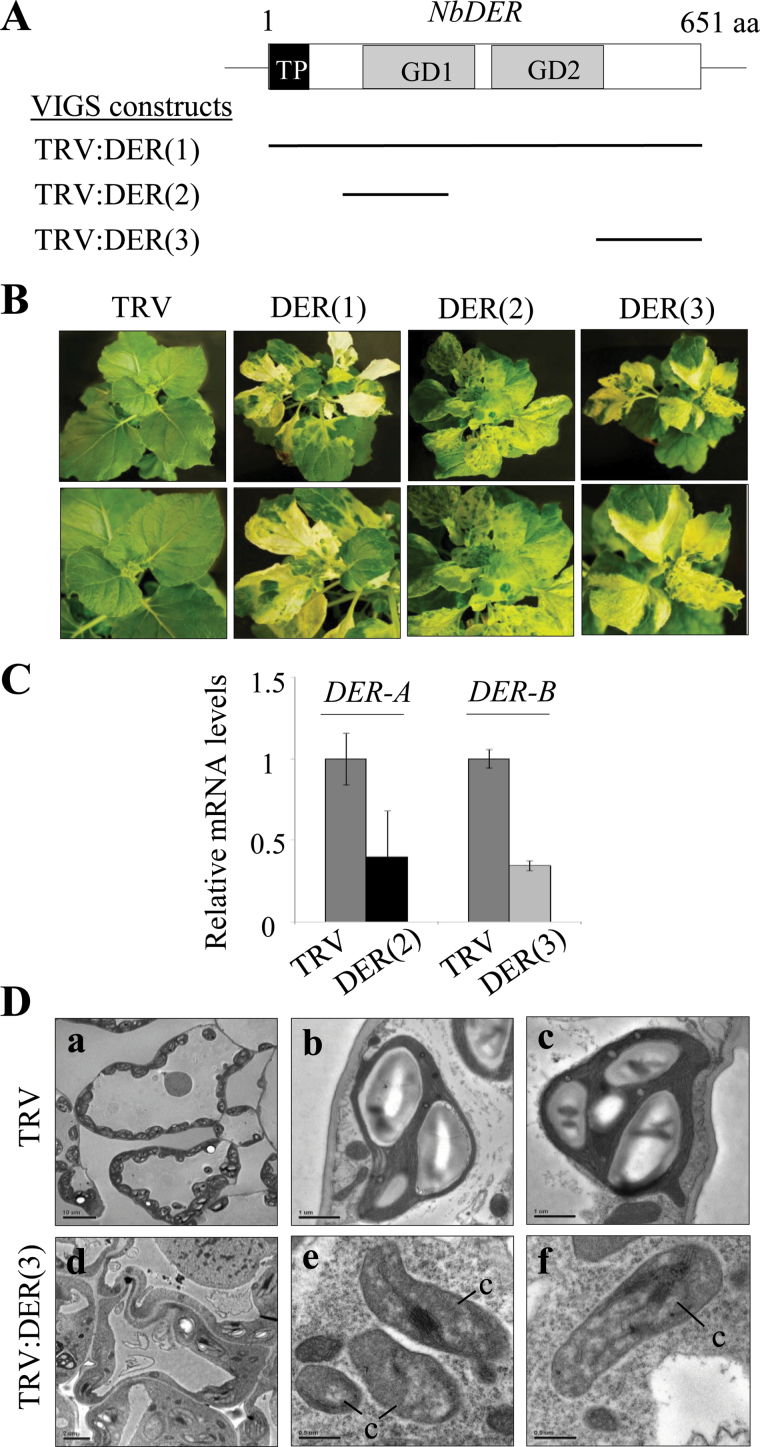
VIGS phenotypes, endogenous NbDER transcript levels, and chloroplast defects. (A) Schematic representation of the NbDER cDNA regions used in the VIGS constructs; the box indicates the protein-coding region of NbDER. The three VIGS constructs are marked by bars; *N. benthamiana* plants were infiltrated with *Agrobacterium* containing the TRV or TRV:DER constructs; aa, amino acids. (B) VIGS phenotypes of TRV control and three TRV:DER VIGS lines; plants were photographed 20 d after infiltration. (C) Real-time quantitative RT-PCR analyses of the NbDER transcript levels in the VIGS lines. DER-A and DER-B primers were used for the analyses; β-tubulin mRNA level was used as a control. (D) Chloroplast ultrastructure; transmission electron micrographs of leaf mesophyll cells (a, d) and chloroplasts (b, c, e, f), from TRV control (a−c) and TRV:DER(3) lines (d−f). c, chloroplasts; bars = 10 μm (a), 2 μm (d), 1 μm (b, c), and 0.5 μm (e, f).

To perform VIGS of NbDER, this work cloned three different NbDER cDNA fragments into the TRV-based VIGS vector pTV00 and infiltrated *N. benthamiana* plants with *Agrobacterium* containing these plasmids (Fig. 3A). TRV:DER(2) and TRV:DER(3) contain a 305-bp and a 402-bp NbDER cDNA fragment, respectively, whereas TRV:DER(1) contains the cDNA corresponding to the full-length coding region. VIGS with each construct resulted in the same leaf-yellowing phenotype without severely affecting overall plant growth and development (Fig. 3B). The colours of the affected leaf sectors in TRV:DER plants varied from pale green to yellow or white. To determine the effect of gene silencing, the amount of endogenous NbDER mRNA in the VIGS lines was measured by real-time quantitative RT-PCR (Fig. 3C). Lower levels of PCR product were amplified in the yellow sectors of TRV:DER(2) and TRV:DER(3) leaves compared with that in TRV control leaves. This result suggests that NbDER is silenced in these VIGS plants.

### Effects of NbDER silencing on chloroplasts

Leaf protoplasts prepared from TRV control and yellow sectors of the TRV:DER(3) lines were visualized by confocal laser scanning microscopy (Supplementary Fig. S5). Chloroplasts in TRV:DER(3) lines were much smaller than those in TRV control: the mean chloroplast diameter in TRV:DER(3) lines was approximately 26% of that of the control (Supplementary Fig. S5A, D). Chlorophyll autofluorescence in TRV:DER(3) protoplasts was not evenly distributed within chloroplasts, and its intensity was reduced to approximately 21% of that of the TRV control (Supplementary Fig. S5A, E). Despite their small size, TRV:DER(3) chloroplasts contained chloroplast nucleoids with significantly increased DAPI fluorescence, whereas TRV control chloroplasts exhibited chloroplast nucleoids with faint DAPI staining (Supplementary Fig. S5B). The ultrastructure of chloroplasts was examined by transmission electron microscopy (Fig. 3D). Transmission electron microscopy of transverse leaf sections in TRV control revealed well-developed chloroplasts with large starch granules (Fig. 3D, panels a–c). By contrast, the yellow sector of TRV:DER(3) leaves contained small and immature chloroplasts with underdeveloped and aggregated thylakoid membranes (Fig. 3D, panels d–f). The abnormal chloroplasts also lacked starch (Fig. 3D, panels e and f), consistent with the lack of black pigment in NbDER VIGS leaves after iodine staining (Supplementary Fig. S5C). These results demonstrate that NbDER deficiency impairs chloroplast biogenesis.

### Defects in accumulation of chloroplast proteins

**Fig. 4.**
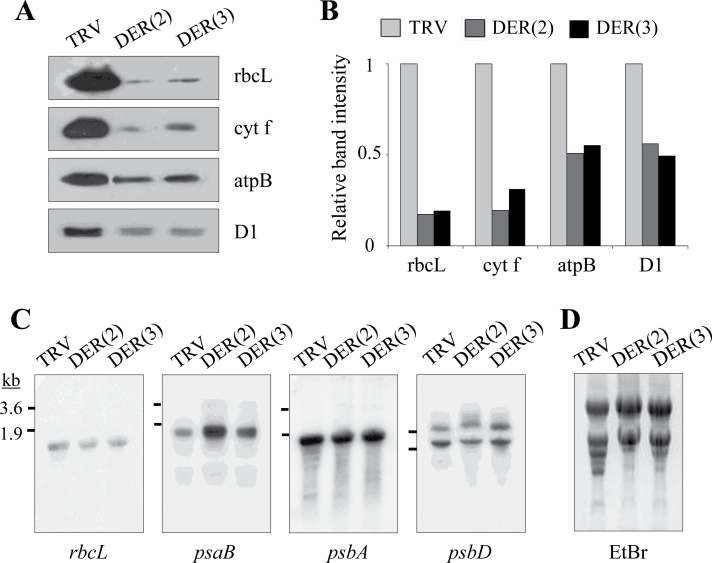
Levels of plastid-encoded proteins and mRNAs. (A, B) Immunoblot analyses of chloroplast proteins (A) and quantification of these data (B); total protein (15 µg) isolated from TRV, TRV:DER(2), and TRV:DER(3) leaves was separated by SDS-PAGE and subjected to immunoblotting using antibodies against rbcL, cyt f, atpB, and D1 (A) and the relative band intensity was calculated by comparison with the band intensity of TRV control (B). (C, D) Transcript accumulation of plastid-encoded genes; RNA gel blot analysis was performed with total RNA (20 µg per lane) isolated from leaves of TRV and TRV:DER lines (C); the ethidium bromide-stained gel shows total rRNA levels (D).

Defects in chloroplast protein accumulation in DER-deficient plants were examined by immunoblot analyses. Equal amounts of total proteins were loaded into each lane for SDS-PAGE. Immunoblotting revealed significantly lower levels of chloroplast proteins rbcL (Rubisco large subunit), cyt f (electron transport protein), atpB (ATP synthase subunit), and D1 (PSII core subunit) in TRV:DER(2) and TRV:DER(3) lines compared with those in TRV control (Fig. 4A, B). To determine if the depletion of the plastid-encoded proteins in TRV:DER plants was caused by defective RNA metabolism, RNA gel blot analysis was carried out with total RNA isolated from TRV and TRV:DER leaves using plastid-encoded genes as probes (Fig. 4C). The ethidium bromide-stained gels of total rRNAs were shown as a control for RNA loading (Fig. 4D). Steady-state transcript levels of plastid-encoded genes including *rbcL*, *psaB*, *psbA*, and *psbD* were not significantly different in TRV:DER lines compared with those in the TRV control (Fig. 4C). This result suggests that the deficiency in chloroplast protein accumulation in TRV:NbDER lines may be caused by defects in translational or post-translational steps.

### Cofractionation of NbDER with the 50S ribosomal subunits

**Fig. 5.**
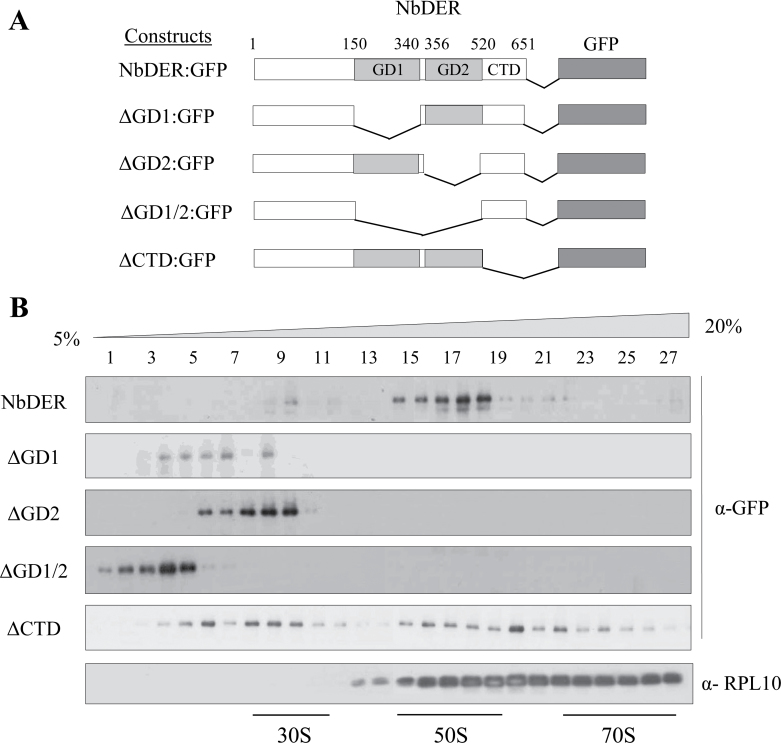
Association of NbDER and its variants with chloroplast ribosomes. (A) Schematic drawings of GFP fusion proteins of NbDER and its variants. (B) NbDER and its variants were expressed in *N. benthamiana* leaves by agroinfiltration; sucrose density gradient fractions were analysed by immunoblotting with anti-GFP and anti-ribosomal protein L10 (RPL10) antibodies; lanes 1–27 indicate the gradient fractions from top (5%) to bottom (20%).

The link between NbDER and chloroplast ribosomes was investigated. The GFP-tagged full-length NbDER and its variants were expressed in *N. benthamiana* leaves by agroinfiltration (Fig. 5A). The leaf cell extract was fractionated using a sucrose density gradient, and the collected fractions were subjected to immunoblotting with anti-GFP antibodies (Fig. 5B). As a control, the same fractions were reacted with anti-RPL10 antibodies that detect the ribosomal protein L10. The full-length NbDER was detected in the fractions containing the 50S subunit, whereas ∆GD1 and ∆GD2 deletion mutants were partially fractionated with the 30S small subunit. ∆GD1/2 mutant protein that lacks both GD1 and GD2 was not incorporated into ribosome fractions. ∆CTD lacking the C-terminal domain was co-fractionated with the 30S small subunit, 50S large subunit, and 70S monosome, suggesting a lack of interaction specificity. These results suggest that NbDER is associated primarily with 50S ribosomal subunits and that the specific association depends on the contribution of multiple domains, including GD1, GD2, and CTD.

### Binding activity of NbDER to ribosomal RNAs

**Fig. 6.**
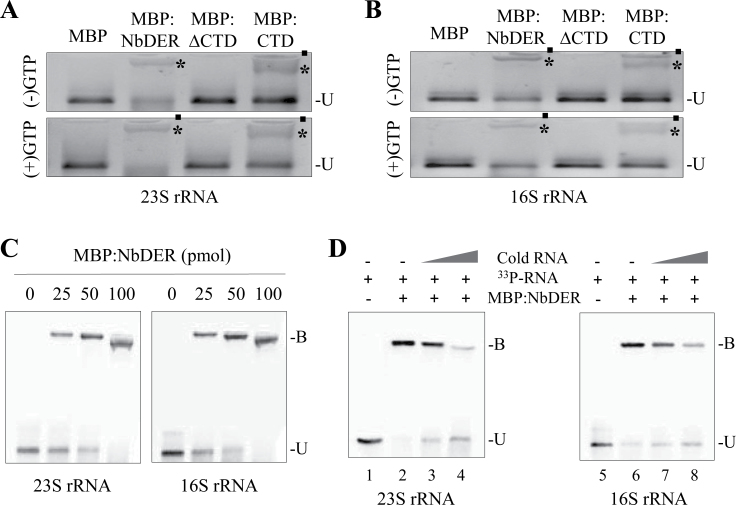
Binding of recombinant NbDER and its variants to 23S and 16S rRNA. (A, B) MBP:NbDER, MBP:∆CTD, and MBP:CTD fusion proteins (75 pmol) were incubated with 200ng of 23S (A) or 16S rRNA (B) in the absence or presence of GTP (100 μM); bound (asterisks) and unbound (U) RNAs were resolved on an agarose gel and visualized by ethidium bromide staining; filled circles indicate the wells of the agarose gel. (C) Increasing concentrations (25, 50, and 100 pmol) of MBP:NbDER fusion proteins were incubated with radiolabelled 23S and 16S rRNAs; bound (B) and unbound (U) RNAs were resolved on an agarose gel and radioactive RNA bands were visualized by PhosphorImager. (D) Gel-mobility shift assays were performed with or without cold competitors; 25 pmol MBP:NbDER fusion protein were incubated with different ratios of radiolabelled and unlabelled 23S and 16S rRNAs; the ratios of radiolabelled RNA:unlabelled RNA are as follows: 1:5 (lane 3), 1:20 (lane 4), 1:5 (lane 7), and 1:20 (lane 8).

The CTD of plant and bacterial DER resembles the K-homology domain, which is an RNA-binding module found in many RNA-binding proteins (Grishin, 2001). This suggests that DER may bind RNA. To test its RNA-binding activity, NbDER (without the transit peptide), NbDER lacking CTD (∆CTD), and CTD alone were fused to maltose-binding protein (MBP), expressed in *E. coli*, and purified for electrophoretic mobility shift assays (Fig. 6 and Supplementary Fig. S6). Because NbDER is associated with the 50S subunit, this work first evaluated binding of these recombinant proteins to 23S rRNA. 23S rRNA synthesized *in vitro* was incubated with MBP:NbDER, MBP:∆CTD, and MBP:CTD, and RNA–protein complexes were resolved by agarose gel electrophoresis. Both MBP:NbDER and MBP:CTD readily formed stable RNA–protein complexes, whereas MBP:∆CTD or MBP alone failed to make the complex (Fig. 6A). MBP:NbDER and MBP:CTD, but not MBP:∆CTD or MBP, also formed RNA–protein complexes with 16S rRNA synthesized *in vitro* (Fig. 6B). Increasing concentrations of MBP:NbDER resulted in increased formation of the RNA–protein complexes (Fig. 6C). EMSA competition assays were performed using MBP:NbDER with different ratios of radiolabelled and unlabelled 23S and 16S rRNAs (Fig. 6D). Excessive amounts of the unlabelled rRNA competitors competed with the ^33^P-labelled rRNAs, suggesting the specificity of the NbDER–rRNA interaction (Fig. 6D). However, none of these recombinant proteins bound to an artificial approximately 160-nucleotide-long RNA derived from the pET-22b(+) vector (Supplementary Fig. S6). These results demonstrate that NbDER binds to both 23S and 16S rRNAs, but not to the sequence-nonspecific RNA.

### Defective rRNA processing in NbDER-deficient chloroplasts

**Fig. 7.**
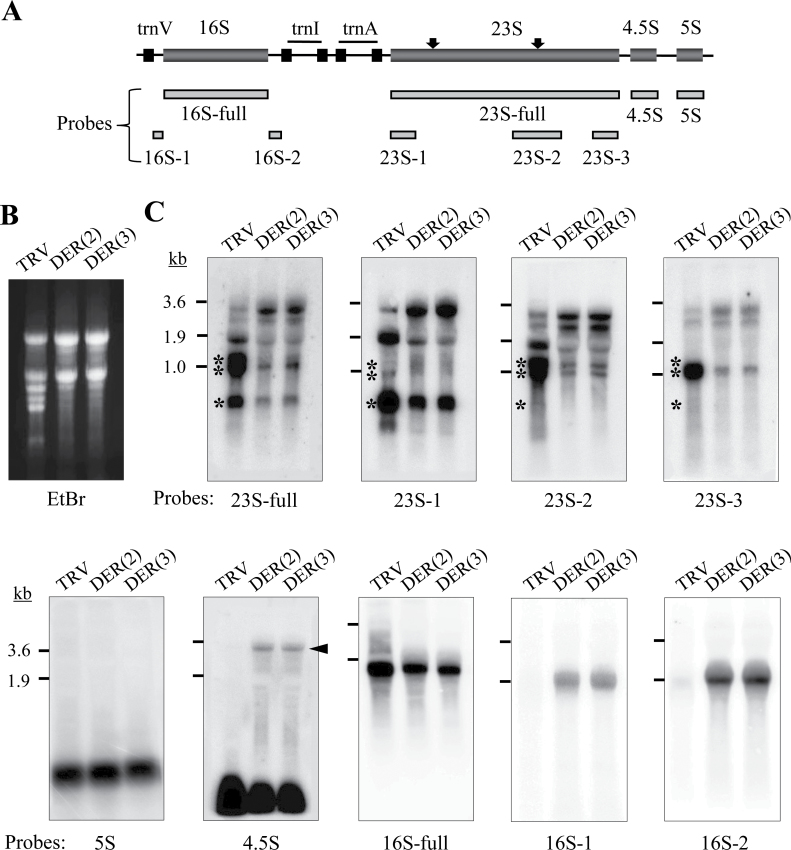
Processing of chloroplast ribosomal RNAs. (A) Schematic representation of rRNAs in the chloroplast rrn operon; dark grey boxes indicate rRNA genes and black boxes indicate tRNA genes; light grey boxes under the operon indicate positions of the PCR-amplified probes; black arrows indicate the cleavage sites of 23S rRNA that produce 0.5-kb, 1.0-kb, and 1.2-kb mature rRNAs. (B, C) RNA gel blot analysis performed with total RNA isolated from leaves of TRV, TRV:DER(2), and TRV:DER(3) lines; ethidium bromide-stained gels show total ribosomal RNAs (B); RNA gel blots (20 μg of total RNA per lane) were hybridized with different probes, as indicated in A (C); asterisks indicate the major forms of mature 23S rRNA; arrowhead indicates 23S–4.5S dicistronic processing intermediate.

Defective rRNA processing is frequently observed when ribosome assembly is impaired. DER depletion in *E. coli* caused accumulation of both 23S and 16S rRNA precursor forms (Hwang and Inouye, 2006). Therefore, this work examined whether rRNA expression or processing was altered in NbDER-depleted chloroplasts by using RNA gel blot analysis (Fig. 7). In chloroplasts of higher plants, the ribosomal RNA (rrn) operon produces a large polycistronic primary transcript (Fig. 7A). The large rRNA precursor form is processed to generate 16S rRNA, a 23S–4.5S rRNA processing intermediate, and 5S rRNA, followed by further processing of the dicistronic 23S–4.5S rRNA intermediate to produce the monocistronic 23S and 4.5S rRNAs. The cleavage of the dicistronic 23S–4.5S rRNA, which requires 3′-end maturation of 4.5S rRNA as a preceding step, is believed to occur after incorporation into ribosomes (Bellaoui *et al.*, 2003). The 16S, 23S, and 5S rRNA precursors undergo additional processing to produce the mature forms; the 23S rRNA is cleaved into 0.5-kb, 1.0-kb, and 1.2-kb mature rRNAs (Kössel *et al.*, 1982; Strittmatter and Kössel, 1984).

RNA gel blot analysis with the 23S-specific probes (23S-full, 23S-1, 23S-2, and 23S-3) revealed a complex pattern of RNA bands for mature and processing forms in the TRV control, of which the 1.2-, 1.0-, and 0.5-kb bands represent the mature forms of 23S rRNA (Fig. 7B, C asterisks). TRV:DER chloroplasts overaccumulated the 23S processing intermediates, whereas accumulation of the mature 23S rRNA forms significantly decreased (Fig. 7C). RNA gel blot analysis with the 4.5S probe revealed accumulation of the 23S–4.5S dicistronic processing intermediate (Fig. 7C arrowhead) in addition to a large amount of normally processed mature 4.5S rRNA in the TRV:DER lines (Fig. 7C), suggesting that the endonucleolytic processing of 4.5S rRNA from the 23S–4.5S dicistronic precursor was affected by DER deficiency, albeit weakly. Next, the probes 16S-1 and 16S-2 recognized the 16S rRNA precursor forms that accumulated in TRV:DER lines, suggesting incomplete processing of pre-16S rRNA at both the 5′ and 3′ ends. The 16S-full probe recognized only mature forms of 16S rRNA in TRV:DER lines, possibly due to much higher levels of mature RNA compared with the levels of precursor RNAs. The 5S probe identified the mature 5S rRNA with no visible accumulation of the precursor forms in the TRV:DER lines (Fig. 7C). These results suggest that DER deficiency results in pleiotropic defects in the processing of chloroplast rRNAs, but primarily disrupts the processing of 23S rRNA.

### Defective polysomal loading of RNA in NbDER-deficient chloroplasts

**Fig. 8.**
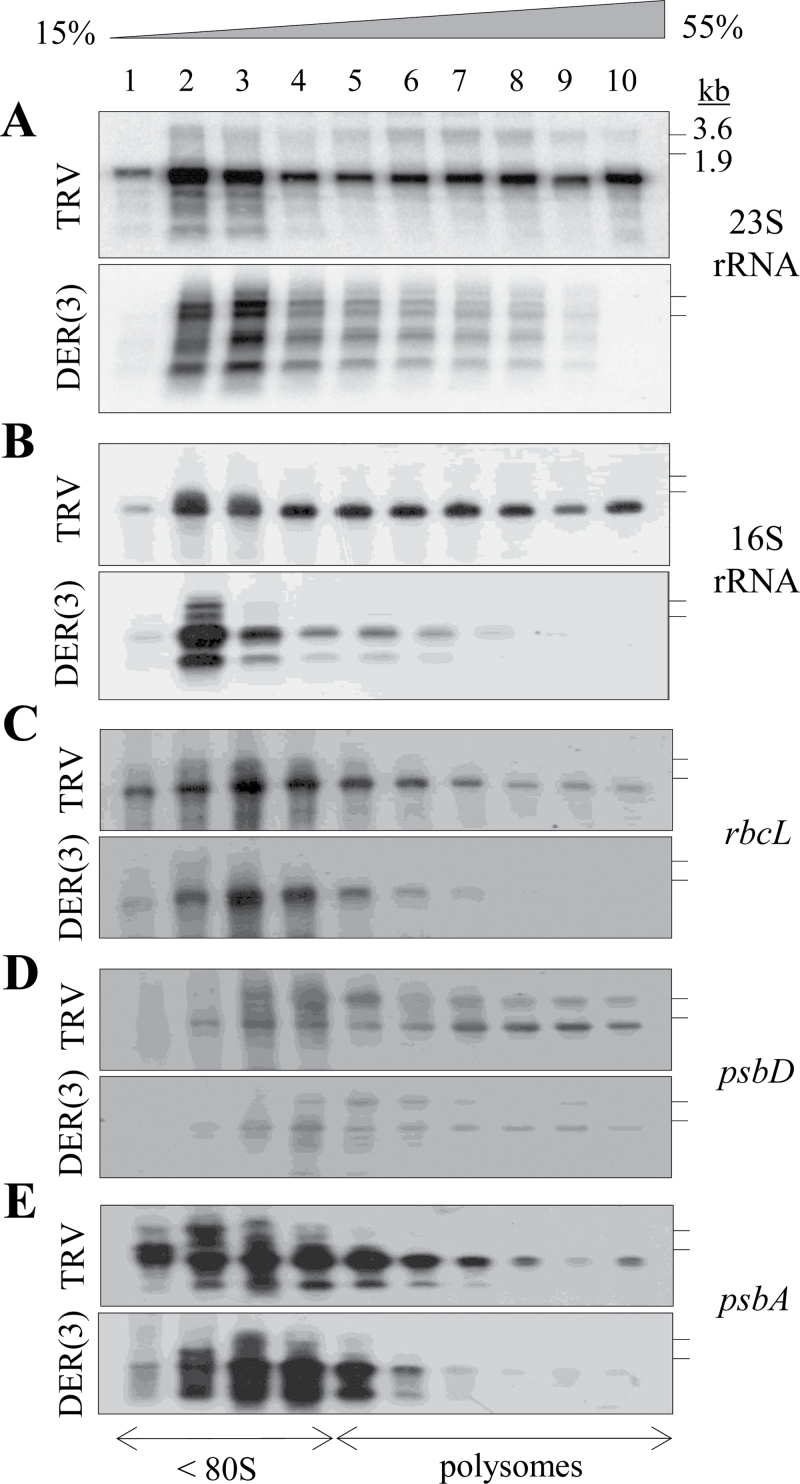
Polysomal loading of chloroplast RNA. Sucrose density gradient fractions from TRV and TRV:DER(3) lines were analysed by RNA gel blot with 23S rRNA, 16S rRNA, rbcL, psbD, and psbA probes. Lanes 1–10 indicate the gradient fractions from top (15%) to bottom (55%). RNA size markers (3.6 and 1.9kb) are indicated in each blot.

Inactivation of DER in *E. coli* inhibited the processing of 50S ribosome assembly, resulting in accumulation of free 30S and 50S ribosomal subunits with a concomitant reduction of 70S monosomes and polysomes (Bharat *et al.*, 2006; Hwang and Inouye, 2006). Because impaired ribosome assembly or rRNA processing often results in abnormal polysomal loading of chloroplast RNAs in plants, this work examined the association of chloroplast RNAs with ribosomes in NbDER-deficient chloroplasts (Fig. 8). Cell extracts from TRV and TRV:DER(3) leaves were sedimented through a 15–55% sucrose density gradient. Fractions were collected after centrifugation, and RNA gel blot analysis of total RNA purified from each gradient fraction was performed using the 23S and 16S rDNA probes. TRV control primarily contained mature 23S and 16S rRNA in the polysome fractions, whereas TRV:DER(3) lines contained high levels of rRNA precursors with substantially reduced amounts of the mature rRNAs in the polysome fractions (Fig. 8A, B). The reduced levels of mature 23S and 16S rRNAs in the polysomal fractions suggest that NbDER-deficient chloroplasts contain fewer polysomes than those in TRV chloroplasts.

The polysomal loading of chloroplast-encoded mRNAs, rbcL, psbD, and psbA, was examined using RNA gel blot analyses with the corresponding probes (Fig. 8C–E). All the mRNAs tested were underrepresented in the polysomal fractions of TRV:DER(3) lines compared with those in TRV, indicating reduced translation of these mRNAs. These results and the observed 23S rRNA processing defects in TRV:DER lines suggest that DER deficiency affects rRNA metabolism, ribosome biogenesis, and consequently translation in chloroplasts.

## Discussion

The DER family GTPases in bacteria and plants are unique among GTPases because they possess two adjacent GTP-binding domains. This work first determined how the two domains contribute to the overall GTPase activity of plant DER. The results suggested that both GTP-binding domains were required for the full GTPase activity of NbDER (Fig. 2). The S162A mutation in GD1 and the S369A mutation in GD2 reduced the GTPase activity to 15.4% and 26.2% of that in controls, and the double mutation of S162A and S369A almost abolished GTPase activity. These results indicate positive cooperativity between these two domains. Consistently, in *E. coli*, S16A and S217A mutations (corresponding to S162A and S369A mutations of *N. benthamiana* DER) in GD1 and GD2 reduced the GTPase activity of DER to 28.6% and 30% of the full activity, respectively (Bharat *et al.*, 2006). Furthermore, *E. coli* DER containing both S16A and S217A mutations could not rescue the lethal phenotype of the conditional *der* null mutant, indicating that GTPase activities of both domains are crucial for its *in vivo* functions (Bharat *et al.*, 2006). However, when *E. coli* cells were grown at high temperature (42 °C), either one of the two GDs of DER was sufficient for cell viability, suggesting that high temperature facilitates a DER-mediated step during ribosome biogenesis (Hwang and Inoue, 2006). The crystal structure of *T. maritima* DER (TmDER) revealed that those serine residues were localized in the G1 motif and positioned in the active site to form hydrogen bonds with two free phosphates of GTP (Robinson *et al.*, 2002).

In this study, computational modelling suggested structural conservation between the plant and bacterial DER GTPases despite their low sequence homology (Supplementary Fig. S3). The 2.5-Å resolution crystal structure of *B. subtilis* YphC (DER)/GDP complex showed that the two GTP-binding domains pack against the K-homology-like C-terminal domain (Muench *et al.*, 2006), consistent with the result from TmDER (Robinson *et al.*, 2002). Changes in the switch II region (G3 motif) associated with nucleotide binding result in dramatic movements of GD1, exposing a positively charged surface that extends over the CTD and GD2. This large, positively charged patch has been implicated in interaction with the negatively charged RNA of the ribosome (Muench *et al.*, 2006). Indeed, *E. coli* DER binds to both 23S and 16S rRNAs *in vitro* (data not shown), consistent with the finding that NbDER binds to both 23S and 16S rRNAs, but not to the vector-driven artificial RNA (Fig. 6 and Supplementary Fig. S6).

Ribosome biogenesis is a very complex procedure that involves a series of events such as rRNA processing, chemical modification of rRNAs and ribosomal proteins, ordered assembly of the ribosomal components, and maturation. *In vitro* reconstitution experiments of ribosome subunits and *in vivo* studies have resulted in an assembly map of the 30S and 50S subunits in *E. coli* (Kaczanowska and Rydén-Aulin, 2007). Although the ribosome subunits can be reconstituted *in vitro* without exogenous factors, the procedure is slow and requires heating steps. In a cell, exogenous factors such as RNA helicases, RNA chaperones, and ribosome-associated GTPases are employed to facilitate ribosome assembly. Chloroplast ribosomes are similar to those of bacteria, consistent with the hypothesis that chloroplasts have endosymbiotic origins. Many ribosome assembly/maturation factors, including the ribosome-associated GTPases, are conserved between chloroplasts and bacteria.

Previous studies in *E. coli* showed that mutations in ribosome-associated GTPases resulted primarily in defects in ribosome assembly and cell death. These mutants commonly showed reduced levels of 70S monosomes in the cell due to improper assembly of individual subunits, indicating that the GTPases provide essential functions for ribosome assembly. DER GTPase interacted specifically with 50S ribosomal subunits and DER deficiency led to accumulation of both 50S and 30S subunits with decreased 70S monosomes and polysomes (Hwang and Inouye, 2006). At low Mg^2+^ concentrations, the *der* mutant contained aberrant 50S subunits that disintegrated into 40S subunits, whereas the assembly of 30S subunits was normal. These data indicate that DER is a novel 50S subunit-associated factor involved in 50S ribosomal biogenesis in *E. coli* (Hwang and Inouye, 2006). The present results suggest that plant and bacterial DER share conserved protein characteristics and functions. Plant DER is predicted to have a very similar protein structure as that of bacterial DER, it is associated primarily with the 50S ribosome subunit, and its depletion leads to defects in chloroplast rRNA processing and polysomal loading of chloroplast RNAs, suggesting a role for plant DER in chloroplast ribosome biogenesis. However, it is still unclear at the molecular level how plant DER participates in ribosome biogenesis in chloroplasts. DER may play a direct role in recruiting ribosomal proteins during ribosome assembly; the yeast Lsg1p ribosome-associated GTPase recruits Rpl10p into the 60S subunit (Hedges *et al.*, 2005). Alternatively, DER may recruit or regulate other assembly factors, such as RNA helicases and rRNA processing factors. Yeast Bsm1p GTPase plays a direct role in recruiting an essential ribosome assembly factor Rcl1 that performs pre-rRNA processing during ribosome maturation (Wegierski *et al.*, 2001). Overexpression of *E. coli* DER suppressed the null phenotype of the *E. coli* rrmJ deletion mutant, which lacks a methyltransferase involved in modification of the U2552 residue of 23S rRNA in the 50S ribosomal subunit (Tan *et al.*, 2002). This genetic evidence implies that DER is a ribosome assembly factor that acts late in biogenesis.

NbDER-depleted chloroplasts exhibited accumulation of the rRNA processing intermediates including the dicistronic 23S–4.5S rRNA precursor and the 16S pre-rRNAs (Fig. 7). Similarly, the *E. coli der* mutant accumulated both 23S and 16S rRNA precursors, although it was suggested that defective 16S rRNA processing was a secondary effect of depleted 50S subunits (Hwang and Inouye, 2006). There is evidence that the final maturation of the chloroplast 23S rRNA occurs after ribosome assembly (Bellaoui *et al.*, 2003; Bollenbach *et al.*, 2005). Ribosomal RNA maturation in *E. coli* appears to depend on translation and it has been proposed that rRNA maturation is part of quality control of newly assembled ribosomes (Srivastava and Schlessinger, 1988; Kaczanowska and Rydén-Aulin, 2007). Defective rRNA processing in chloroplasts was observed in response to mutations in ribonucleases that were directly involved in rRNA maturation, such as polynucleotide phosphorylase and RNase R homologue 1 (Walter *et al.*, 2002; Bollenbach *et al.*, 2005). However, mutations that affected ribosome assembly or translation (e.g. a mutation in RNC1 encoding a splicing factor for chloroplast ribosomal protein mRNAs and tRNAs) also resulted in accumulation of rRNA processing intermediates (Watkins *et al.*, 2007). Further investigations are required to determine whether rRNA maturation and protein synthesis are interdependent in chloroplasts.

It is intriguing that many ribosome-associated GTPases, including DER, function late in the biogenesis process (Britton, 2009; Verstraeten *et al.*, 2011). It has been proposed that these GTPases may serve as internal checkpoints by linking ribosome assembly with intracellular GTP concentrations (Britton, 2009). Low GTP levels are associated with nutrient deficiency; therefore, by sensing GTP levels, the ribosome-associated GTPases may sense nutrient or energy stress and halt further ribosome assembly. When the availability of amino acids decreases under a threshold level, a complex cellular process termed the stringent response becomes activated in *E. coli* (Chatterji and Ojha, 2001). The hallmark of the stringent response is the accumulation of the second messenger (p)ppGpp, which leads to rapid transcriptional inhibition of rRNA and ribosomal protein genes (Magnusson *et al.*, 2005). Overexpression of (p)ppGpp suppresses the growth defect of the *E. coli der* (N321D) mutant, indicating that (p)ppGpp may regulate DER function under stress conditions (Hwang and Inouye, 2008). This result is intriguing, considering that the bacterial ppGpp is identified in chloroplasts of plant cells (Takahashi *et al.*, 2004). ppGpp levels in chloroplasts increase dramatically in response to diverse abiotic and biotic stresses. Furthermore, addition of ppGpp inhibits chloroplast RNA polymerase activity *in vitro*, suggesting the existence of a bacterial-type stringent response in plants (Sato *et al.*, 2009). It will be interesting to determine whether plant DER plays a role in rapid regulation of ribosome assembly in response to elevated ppGpp levels in chloroplasts.

## Supplementary material

Supplementary data are available at *JXB* online.

Supplementary Fig. S1. Protein structure of NbDER and sequence alignment with plant and bacterial DER proteins.

Supplementary Fig. S2. Comparison of GD1 and GD2 sequences among DER homologues.

Supplementary Fig. S3. Computational modelling of plant DER.

Supplementary Fig. S4. An embryonic lethal phenotype of the *Arabidopsis der* mutant.

Supplementary Fig. S5. Chloroplast defects in TRV:DER VIGS lines.

Supplementary Fig. S6. Gel-mobility shift assays of recombinant NbDER and its variants.

Supplementary Data

## References

[CIT0001] Ahnn J March PE Takiff HE Inouye M 1986 A GTP-binding protein of *Escherichia coli* has homology to yeast RAS proteins. Proceedings of the National Academy of Sciences, USA 83, 8849–8853 10.1073/pnas.83.23.8849 PMC387030 3097637

[CIT0002] Bang WY Chen J Jeong IS 2012 Functional characterization of ObgC in ribosome biogenesis during chloroplast development. The Plant Journal 71, 122–134 22380942 10.1111/j.1365-313X.2012.04976.x

[CIT0003] Barkan A 1993 Nuclear mutants of maize with defects in chloroplast polysome assembly have altered chloroplast RNA metabolism. The Plant Cell 5, 389–402 12271069 10.1105/tpc.5.4.389 PMC160279

[CIT0004] Bellaoui M Keddie JS Gruissem W 2003 DCL is a plant-specific protein required for plastid ribosomal RNA processing and embryo development. Plant Molecular Biology 53, 531–543 15010617 10.1023/B:PLAN.0000019061.79773.06

[CIT0005] Bharat A Jiang M Sullivan SM Maddock JR Brown ED 2006 Cooperative and critical roles for both G domains in the GTPase activity and cellular function of ribosome-associated *Escherichia coli* EngA. Journal of Bacteriology 188, 7992–7996 16963571 10.1128/JB.00959-06 PMC1636305

[CIT0006] Bollenbach TJ Lange H Gutierrez R Erhardt M Stern DB Gagliardi D 2005 RNR1, a 3′-5′ exoribonuclease belonging to the RNR superfamily, catalyzes 3′ maturation of chloroplast ribosomal RNAs in *Arabidopsis thaliana* . Nucleic Acids Research 33, 2751–2763 15891117 10.1093/nar/gki576 PMC1110743

[CIT0007] Britton RA 2009 Role of GTPases in bacterial ribosome assembly. Annual Review of Microbiology 63, 155–176 10.1146/annurev.micro.091208.073225 19575570

[CIT0008] Caldon CE March PE 2003 Function of the universally conserved bacterial GTPases. Current Opinion in Microbiology 6, 135–139 12732302 10.1016/s1369-5274(03)00037-7

[CIT0009] Chatterji D Ojha AK 2001 Revisiting the stringent response, ppGpp and starvation signaling. Current Opinion in Microbiology 4, 160–165 11282471 10.1016/s1369-5274(00)00182-x

[CIT0010] Chen X Court DL Ji X 1999 Crystal structure of ERA: a GTPase-dependent cell cycle regulator containing an RNA binding motif. Proceedings of the National Academy of Sciences, USA 96, 8396–8401 10.1073/pnas.96.15.8396 PMC17527 10411886

[CIT0011] Cho HS Lee SS Kim KD Kim SJ Hwang I Lim JS Park YI Pai H-S 2004 DNA gyrase is involved in chloroplast nucleoid partitioning. The Plant Cell 16, 2665–2682 15367714 10.1105/tpc.104.024281 PMC520963

[CIT0012] Grishin NV 2001 KH domain: one motif, two folds. Nucleic Acids Research 29, 638–643 11160884 10.1093/nar/29.3.638 PMC30387

[CIT0013] Hedges J West M Johnson AW 2005 Release of the export adapter, Nmd3p, from the 60S ribosomal subunit requires Rpl10p and the cytoplasmic GTPase Lsg1p. EMBO Journal 24, 567–579 15660131 10.1038/sj.emboj.7600547 PMC548654

[CIT0014] Hwang J Inouye M 2006 The tandem GTPase, Der, is essential for the biogenesis of 50S ribosomal subunits in *Escherichia coli* . Molecular Microbiology 61, 1660–1672 16930151 10.1111/j.1365-2958.2006.05348.x

[CIT0015] Hwang J Inouye M 2008 RelA functionally suppresses the growth defect caused by a mutation in the G domain of the essential Der protein. Journal of Bacteriology 190, 3236–3243 18296517 10.1128/JB.01758-07 PMC2347370

[CIT0016] Im CH Hwang SM Son YS Heo JB Bang WY Suwastika IN Shiina T Bahk JD 2011 Nuclear/nucleolar GTPase 2 proteins as a subfamily of YlqF/YawG GTPases function in pre-60S ribosomal subunit maturation of mono- and dicotyledonous plants. Journal of Biological Chemistry 286, 8620–8632 21205822 10.1074/jbc.M110.200816 PMC3048744

[CIT0017] Ingram GC Simon R Carpenter R Coen ES 1998 The *Antirrhinum* ERG gene encodes a protein related to bacterial small GTPases and is required for embryonic viability. Current Biology 8, 1079–1082 9768362 10.1016/s0960-9822(98)70445-2

[CIT0018] Jeon Y Jung HJ Kang H Park YI Lee SH Pai HS 2012 S1 domain containing STF modulates plastid transcription and chloroplast biogenesis in *Nicotiana benthamiana* . New Phytologist 193, 349–363 22050604 10.1111/j.1469-8137.2011.03941.x

[CIT0019] Jiang M Datta K Walker A Strahler J Bagamasbad P Andrews PC Maddock JR 2006 The *Escherichia coli* GTPase CgtAE is involved in late steps of large ribosome assembly. Journal of Bacteriology 188, 6757–6570 16980477 10.1128/JB.00444-06 PMC1595513

[CIT0020] Kaczanowska M Rydén-Aulin M 2007 Ribosome biogenesis and the translation process in *Escherichia coli* . Microbiology and Molecular Biology Reviews 71, 477–494 17804668 10.1128/MMBR.00013-07 PMC2168646

[CIT0021] Kang YW Lee JY Jeon Y Kim M Cheong GW Pai H-S 2010 *In vivo* effects of NbSiR silencing on chloroplast development in *Nicotiana benthamiana* . Plant Molecular Biology 72, 569–583 20047069 10.1007/s11103-009-9593-8

[CIT0022] Kössel H Edwards K Koch W Langridge P Schiefermayr E Schwarz Z Strittmatter G Zenke G 1982 Structural and functional analysis of an rRNA operon and its flanking tRNA genes from *Zea mays* chloroplasts. Nucleic Acids Symposium Series 11, 117–120 7183954

[CIT0023] Kwon KC Cho MH 2008 Deletion of the chloroplast-localized AtTerC gene product in *Arabidopsis thaliana* leads to loss of the thylakoid membrane and to seedling lethality. The Plant Journal 55, 428–442 18429937 10.1111/j.1365-313X.2008.03523.x

[CIT0024] Liu H Lau E Lam MP 2010 OsNOA1/RIF1 is a functional homolog of AtNOA1/RIF1: implication for a highly conserved plant cGTPase essential for chloroplast function. New Phytologist 187, 83–105 20456051 10.1111/j.1469-8137.2010.03264.x

[CIT0025] Magnusson LU Farewell A Nyström T 2005 ppGpp: a global regulator in *Escherichia coli* . Trends in Microbiology 13, 236–242 15866041 10.1016/j.tim.2005.03.008

[CIT0026] Majeran W Friso G Asakura Y Qu X Huang M Ponnala L Watkins KP Barkan A van Wijk KJ 2012 Nucleoid-enriched proteomes in developing plastids and chloroplasts from maize leaves: a new conceptual framework for nucleoid functions. Plant Physiology 158, 156–189 22065420 10.1104/pp.111.188474 PMC3252073

[CIT0027] Minkovsky N Zarimani A Chary VK Johnstone BH Powell BS Torrance PD Court DL Simons RW Piggot PJ 2002 Bex, the *Bacillus subtilis* homolog of the essential *Escherichia coli* GTPase Era, is required for normal cell division and spore formation. Journal of Bacteriology 184, 6389–6394 12399511 10.1128/JB.184.22.6389-6394.2002 PMC151948

[CIT0028] Muench SP Xu L Sedelnikova SE Rice DW 2006 The essential GTPase YphC displays a major domain rearrangement associated with nucleotide binding. Proceedings of the National Academy of Sciences, USA 103, 12359–12364 10.1073/pnas.0602585103 PMC1567884 16894162

[CIT0029] Pfalz J Liere K Kandlbinder A Dietz KJ Oelmüller R 2006 pTAC-2, -6, and -12 are components of the transcriptionally active plastid chromosome that are required for plastid gene expression. The Plant Cell 18, 176–197 16326926 10.1105/tpc.105.036392 PMC1323492

[CIT0030] Robinson VL Hwang J Fox E Inouye M Stock AM 2002 Domain arrangement of Der, a switch protein containing two GTPase domains. Structure 10, 1649–1658 12467572 10.1016/s0969-2126(02)00905-x

[CIT0031] Sato A Kobayashi G Hayashi H Yoshida H Wada A Maeda M Hiraga S Takeyasu K Wada C 2005 The GTP binding protein Obg homolog ObgE is involved in ribosome maturation. Genes to Cells 10, 393–408 15836769 10.1111/j.1365-2443.2005.00851.x

[CIT0032] Sato M Takahashi K Ochiai Y Hosaka T Ochi K Nabeta K 2009 Bacterial alarmone, guanosine 5′-diphosphate 3′-diphosphate (ppGpp), predominantly binds the beta′ subunit of plastid-encoded plastid RNA polymerase in chloroplasts. Chembiochem 10, 1227–1233 19308923 10.1002/cbic.200800737

[CIT0033] Sekine K Fujiwara M Nakayama M Takao T Hase T Sato N 2007 DNA binding and partial nucleoid localization of the chloroplast stromal enzyme ferredoxin:sulfite reductase. FEBS Journal 274, 2054–2069 17371503 10.1111/j.1742-4658.2007.05748.x

[CIT0034] Sharma MR Barat C Wilson DN Booth TM Kawazoe M Hori-Takemoto C Shirouzu M Yokoyama S Fucini P Agrawal RK 2005 Interaction of Era with the 30S ribosomal subunit implications for 30S subunit assembly. Molecular Cell 18, 319–329 15866174 10.1016/j.molcel.2005.03.028

[CIT0035] Srivastava AK Schlessinger D 1988 Coregulation of processing and translation: mature 5′ termini of *Escherichia coli* 23S ribosomal RNA form in polysomes. Proceedings of the National Academy of Sciences, USA 85, 7144–7148 10.1073/pnas.85.19.7144 PMC282140 3050989

[CIT0036] Strittmatter G Kössel H 1984 Cotranscription and processing of 23S, 4.5S and 5S rRNA in chloroplasts from *Zea mays* . Nucleic Acids Research 12, 7633–7647 6093045 10.1093/nar/12.20.7633 PMC320190

[CIT0037] Takahashi K Kasai K Ochi K 2004 Identification of the bacterial alarmone guanosine 5′-diphosphate 3′-diphosphate (ppGpp) in plants. Proceedings of the National Academy of Sciences, USA 101, 4320–4324 10.1073/pnas.0308555101 PMC384739 15010537

[CIT0038] Tan J Jakob U Bardwell JC 2002 Overexpression of two different GTPases rescues a null mutation in a heat-induced rRNA methyltransferase. Journal of Bacteriology 184, 2692–2698 11976298 10.1128/JB.184.10.2692-2698.2002 PMC135011

[CIT0039] Tzafrir I Pena-Muralla R Dickerman A 2004 Identification of genes required for embryo development in *Arabidopsis* . Plant Physiology 135, 1206–1220 15266054 10.1104/pp.104.045179 PMC519041

[CIT0040] Verstraeten N Fauvart M Versées W Michiels J 2011 The universally conserved prokaryotic GTPases. Microbiology and Molecular Biology Reviews 75, 507–542 21885683 10.1128/MMBR.00009-11 PMC3165542

[CIT0041] Voinnet O Rivas S Mestre P Baulcombe D 2003 An enhanced transient expression system in plants based on suppression of gene silencing by the p19 protein of tomato bushy stunt virus. The Plant Journal 33, 949–956 12609035 10.1046/j.1365-313x.2003.01676.x

[CIT0042] Walter M Kilian J Kudla J 2002 PNPase activity determines the efficiency of mRNA 3′-end processing, the degradation of tRNA and the extent of polyadenylation in chloroplasts. EMBO Journal 21, 6905–6914 12486011 10.1093/emboj/cdf686 PMC139106

[CIT0043] Watkins KP Kroeger TS Cooke AM Williams-Carrier RE Friso G Belcher SE van Wijk KJ Barkan A 2007 A ribonuclease III domain protein functions in group II intron splicing in maize chloroplasts. The Plant Cell 19, 2606–2623 17693527 10.1105/tpc.107.053736 PMC2002627

[CIT0044] Wegierski T Billy E Nasr F Filipowicz W 2001 Bms1p, a G-domain-containing protein, associates with Rcl1p and is required for 18S rRNA biogenesis in yeast. RNA 7, 1254–1267 11565748 10.1017/s1355838201012079 PMC1370170

[CIT0045] Williams PM Barkan A 2003 A chloroplast-localized PPR protein required for plastid ribosome accumulation. The Plant Journal 36, 675–686 14617068 10.1046/j.1365-313x.2003.01915.x

