# Supplementary material for: DER containing two consecutive GTP-binding domains plays an essential role in chloroplast ribosomal RNA processing and ribosome biogenesis in higher plants
Source: J Exp Bot. 2013 Nov 23;65(1):117–30. doi: 10.1093/jxb/ert360 (PMC3883289; doi:10.1093/jxb/ert360)
Supplement: Supplementary Data [file supp_65_1_117__index.html]

DER containing two consecutive GTP-binding domains plays an essential role in chloroplast ribosomal RNA processing and ribosome biogenesis in higher plants — DER containing two consecutive GTP-binding domains plays an essential role in chloroplast ribosomal RNA processing and ribosome biogenesis in higher plants — Supplementary Data 

# DER containing two consecutive GTP-binding domains plays an essential role in chloroplast ribosomal RNA processing and ribosome biogenesis in higher plants

## Supplementary Data

Data files

**Files in this Data Supplement:**

- Supplementary Data - Supplementary Data
- Supplementary Data - Supplementary Data
