# Supplementary material for: DER containing two consecutive GTP-binding domains plays an essential role in chloroplast ribosomal RNA processing and ribosome biogenesis in higher plants
Source: J Exp Bot. 2013 Nov 23;65(1):117–30. doi: 10.1093/jxb/ert360 (PMC3883289; doi:10.1093/jxb/ert360)
Supplement: Supplementary Data [file supp_ert360_jexbot103218_file001.pdf]

Supplementary Figure S1

A

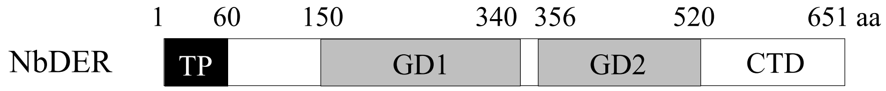

B

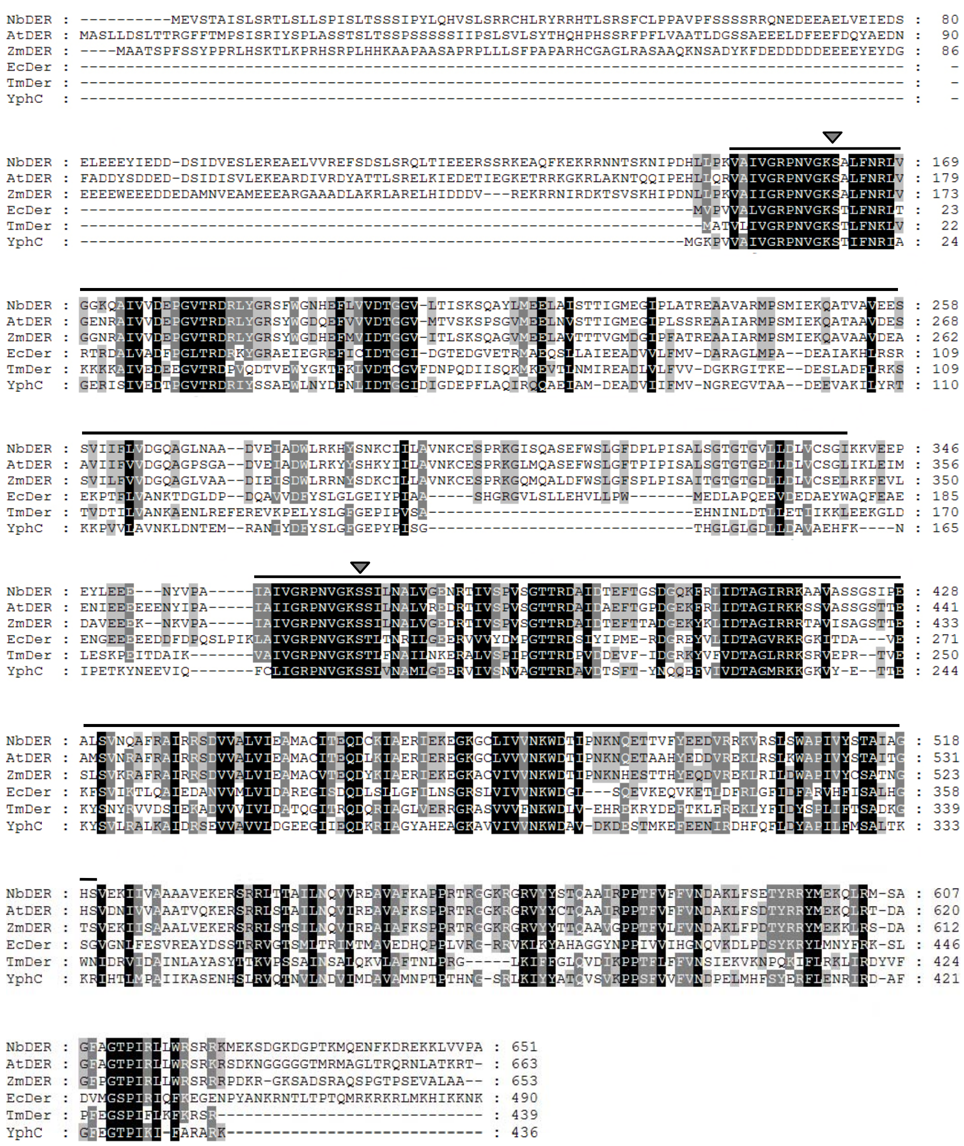

Supplementary Fig. S1. Protein structure of NbDER and sequence alignment with plant and bacterial DER proteins.

(A) NbDER protein structure showing the transit peptide (TP), two consecutive GTP-binding domains (GD1 and GD2), and KH-like C-terminal domain (CTD). aa, amino acids.

(B) Amino acid sequence alignment of NbDER and its homologs from *Arabidopsis thaliana* (AtDER; At3g12080), *Zea maize* (ZmDER; ACL53683), *Escherichia coli* (EcDER; AAC75564), *Thermotoga maritima* (TmDER; AAD36514), and *Bacillus subtilis* (YphC; AAC83966). The conserved residues are *boxed* in *black* or *light gray* based on the degree of conservation, respectively. The *overlines* indicate the two GTP-binding domains. The crucial Ser162 and Ser369 residues are marked with *arrowheads*.

Supplementary Figure S2

A

```
              G1                  G2                 G3
GD1-Nb : VAIVGRPNVGKSALFNRLVGGKQAIVVDEPGVTRDRLYGRSFWGNHEFLVVDTGGVLTISKSQAYLMEELAISTTI : 226
GD1-At : VAIVGRPNVGKSALFNRLVGENRAIVVDEPGVTRDRLYGRSYWGDQEFVVVDTGGVMTVSKSPSGVMEELNVSTTI : 236
GD1-Zm : VAIIGRPNVGKSALFNRLVGGNRAIVVDEPGVTRDRLYGRSYWGDHEFMVIDTGGVITLSKSQAGVMEELAVTTTV : 230
GD1-Ec : VALVGRPNVGKSTLFNRLTRTRDALVADFPGLTRDRKYGRAEIEGREFICIDTGGI-DGTEDG------------- : 66
GD1-Tm : VLIVGRPNVGKSTLFNKLVKKKKAIVEDEEGVTRDPVQDTVEWYGKTFKLVDTCGVFDNPQDI------------- : 66
GD1-Bs : VAIVGRPNVGKSTIFNRIAGERISIVEDTPGVTRDRIYSSAEWLNYDFNLIDTGGI-DIGDEP------------- : 67

                                                                         G4
GD1-Nb : GMEGIPLATREAAVARMPSMIEKQATVAVEESSVIIFLVDGQAGLNAADVEIADWLRKHYSNKCIILAVNKCESPR : 302
GD1-At : GMEGIPLSSREAAIARMPSMIEKQATAAVDESAVIIFVVDGQAGPSGADVEIADWLRKYYSHKYIILAVNKCESPR : 312
GD1-Zm : GMDGIPFATREAAIARMPSMIEKQAVAAVDEASVILFVVDGQAGLVAADIEISDWLRRNYSDKCILLAVNKCESPR : 306
GD1-Ec : -----------------VETRMAEQSLLAIEEADVVLFMVDARAGLMPADEAIAKHLRS--REKPTFLVANKTDGLD : 124
GD1-Tm : -----------------ISQKMKEVTLNMIREADLVLFVVDGKRGITKEDESLADFLRK--STVDTILVANKAENLR : 124
GD1-Bs : -----------------FLAQIRQQAEIAMDEADVIIFMVNGREGVTAADEEVAKILYR--TKKPVVLAVNKLDNTE : 125

                    G5
GD1-Nb : KGISQAS-EFWSLGF-DPLPISALSGTGTGVLLDLVCSGI : 340
GD1-At : KGLMQAS-EFWSLGF-TPIPISALSGTGTGELLDLVCSGL : 350
GD1-Zm : KGQMQAL-DFWSLGF-SPLPISAITGTGTGDLLDLVCSEL : 344
GD1-Ec : PDQAVV--DFYSLGLGEIYPIAASHGRGVLSLLEHVL--- : 159
GD1-Tm : EFEREVKPELYSLGFGEPIPVSAEHNINLDTLLETIIKKL : 164
GD1-Bs : MRANIY--DFYSLGFGEPYPISGTHGLGLGDLLDAVAEHF : 163
```

B

```
                 G1                  G2                  G3
GD2-Nb : ----IAIVGRPNVGKSSILNALVGENRTIVSPVSGTTRDAIDTEFTGSDGQKFRLIDTAGIRRKAAVASSGSIPEALSVN : 433
GD2-At : --------GRPNVGKSSILNALVREDRTIVSPVSGTTRDAIDAEFTGPDGEKFRLIDTAGIRKKSSVASSGSTTEAMSVN : 446
GD2-Zm : KVPAIAIVGRPNVGKSSILNALVGEDRTIVSPVSGTTRDAIDTEFTTADGEKYKLIDTAGIRRRTAVISAGSTTESLSVK : 438
GD2-Ec : -PIKLAIVGRPNVGKSTLTNRILGEERVVVYDMPGTTRDSIYIPME-RDGREYVLIDTAGVRKRGKI--T-DAVEKFSVI : 276
GD2-Tm : DAIKVAIVGRPNVGKSTLFNAILNKERALVSPIPGTTRDPVDDEVF-IDGRKYVFVDTAGLRRKSRV--EPRTVEKYSNY : 255
GD2-Bs : ----FCLIGRPNVGKSSLVNAMLGEERVIVSNVAGTTRDAVDTSFT-YNQQEFVIVDTAGMRKKGKV--Y-ETTEKYSVL : 249

                                                         G4                             G5
GD2-Nb : QAFRAIRRSDVVALVIEAMACITEQDCKIAERIEKEGKGCLIVVNKWDTIPNKNQETTVFYEEDVRRKVRSLSWAPIVYS : 513
GD2-At : RAFRAIRRSDVVALVIEAMACITEQDLKIAERIEREGKGCLVVVNKWDTIPNKNQETAAHYEDDVREKLRSLKWAPIVYS : 526
GD2-Zm : RAFRAIRRSDVVALVIEAMACVTEQDYKIAERIEKEGKACVIVVNKWDTIPNKNHESTTHYEQDVREKLRILDWAPIVYC : 518
GD2-Ec : KTLQAIEDANVVMLVIDAREGISDQDLSLLGFILNSGRSLVIVVNKWDGL-SQEVK--EQVKETLDFRLGFIDFARVHFI : 353
GD2-Tm : RVVDSIEKADVVVIVLDATQGITRQDQRIAGLVERRGRASVVVFNKWDLV-EHREKRYDEFTKLFREKLYFIDYSPLIFT : 334
GD2-Bs : RALKAIDRSEVVAVVLDGEEGIIEQDKRIAGYAHEAGKAVVIVVNKWDAV-DKDESTMKEFEENIRDHFQFLDYAPILFM : 328

         _
GD2-Nb : TAIAGH------------- : 519
GD2-At : TAITGHSVDNIVVAAATV- : 544
GD2-Zm : SATNGTSVEKIISAAALV- : 536
GD2-Ec : SALHGSGVGNLFESVREAY : 372
GD2-Tm : SADKGWNIDRVIDAINLAY : 353
GD2-Bs : SALTKKRIHTLMPAIIKA- : 347
```

Supplementary Fig. S2. Comparison of GD1 and GD2 sequences among DER homologs.

(A) Amino acid sequences of GD1 from NbDER (GD1-Nb) and its homologs from *Arabidopsis* (GD1-At), *Z. maize* (GD1-Zm), *E. coli* (GD1-Ec), *T. maritima* (GD1-Tm), and *B. subtilis* (GD1-Bs) were aligned. The GTPase motifs (G1–G5) marked with *overlines* were defined according to Muench *et al.* (2006). The G motifs (G1–G5) are well conserved between plant and prokaryotic DER proteins; G1 is involved in binding of the phosphates of GTP and GDP; G2 shows large structural differences between the GTP- and GDP-bound states, and thus is called the switch I region; G3 is involved in Mg^{2+} coordination and binding to the γ-phosphate, and is called the switch II region; G4 determines nucleotide specificity by forming hydrogen bonds with guanine rings; and G5 interacts with guanine, but is not well conserved across GTPases (Verstraeten et al., 2011).

(B) Amino acid sequence alignment of GD2 from various DER proteins.

Supplementary Figure S3

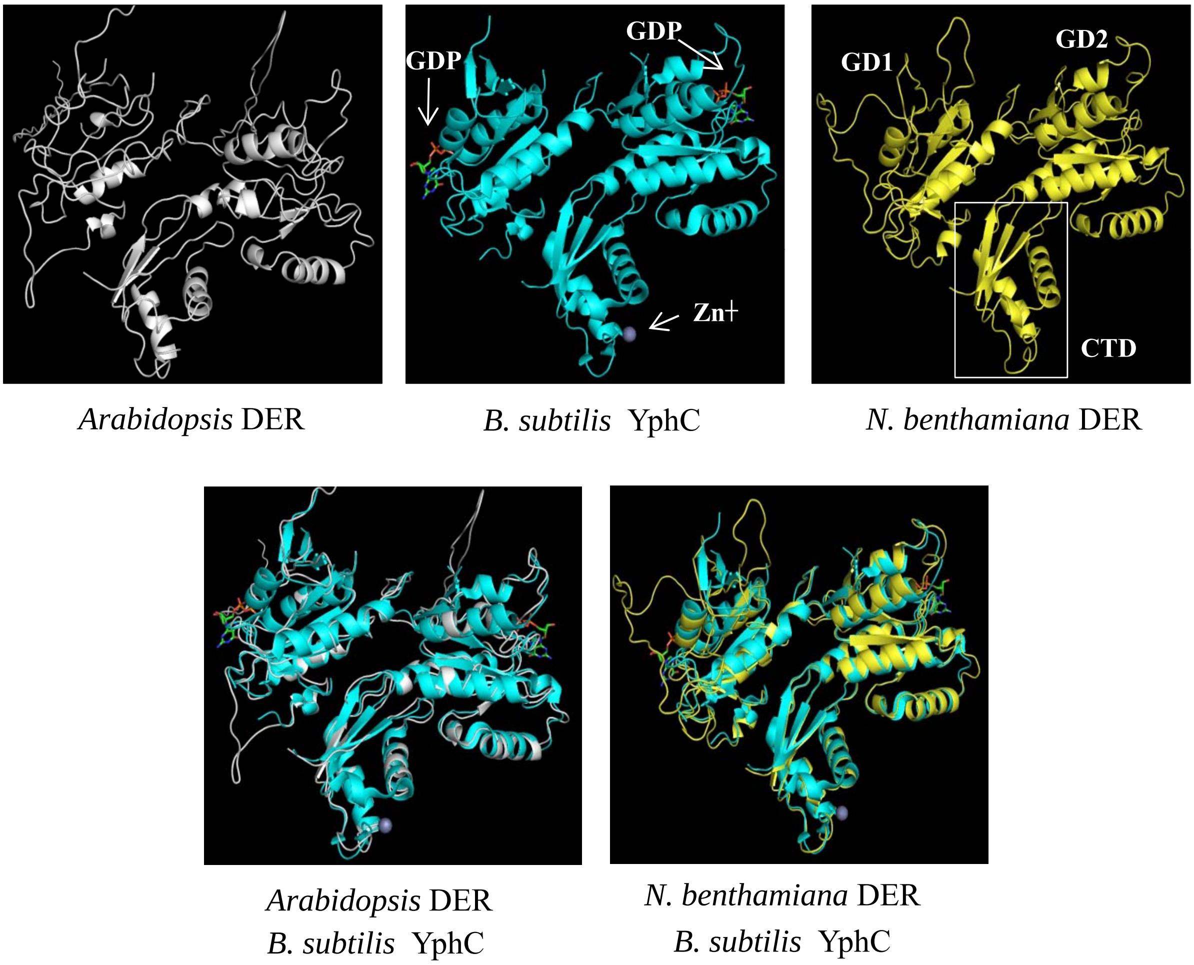

Supplementary Fig. S3. Computational modeling of plant DER.

The tertiary structure of DER proteins of *N. benthamiana* and *Arabidopsis* was predicted using the automated homology modeling server [(PS)2: Protein Structure Prediction Server; http://ps2.life.nctu.edu.tw/] with *B. subtilis* YphC with bound GDP in both GTP-binding domains as template. The predicted molecular model is edited using PyMOL molecular graphics system (version 1.1). The GD1, GD2, and CTD are marked. Although the CTD of plant DER shows only limited sequence homology, its overall structure is similar to that of prokaryotic DER proteins.

Supplementary Figure S4

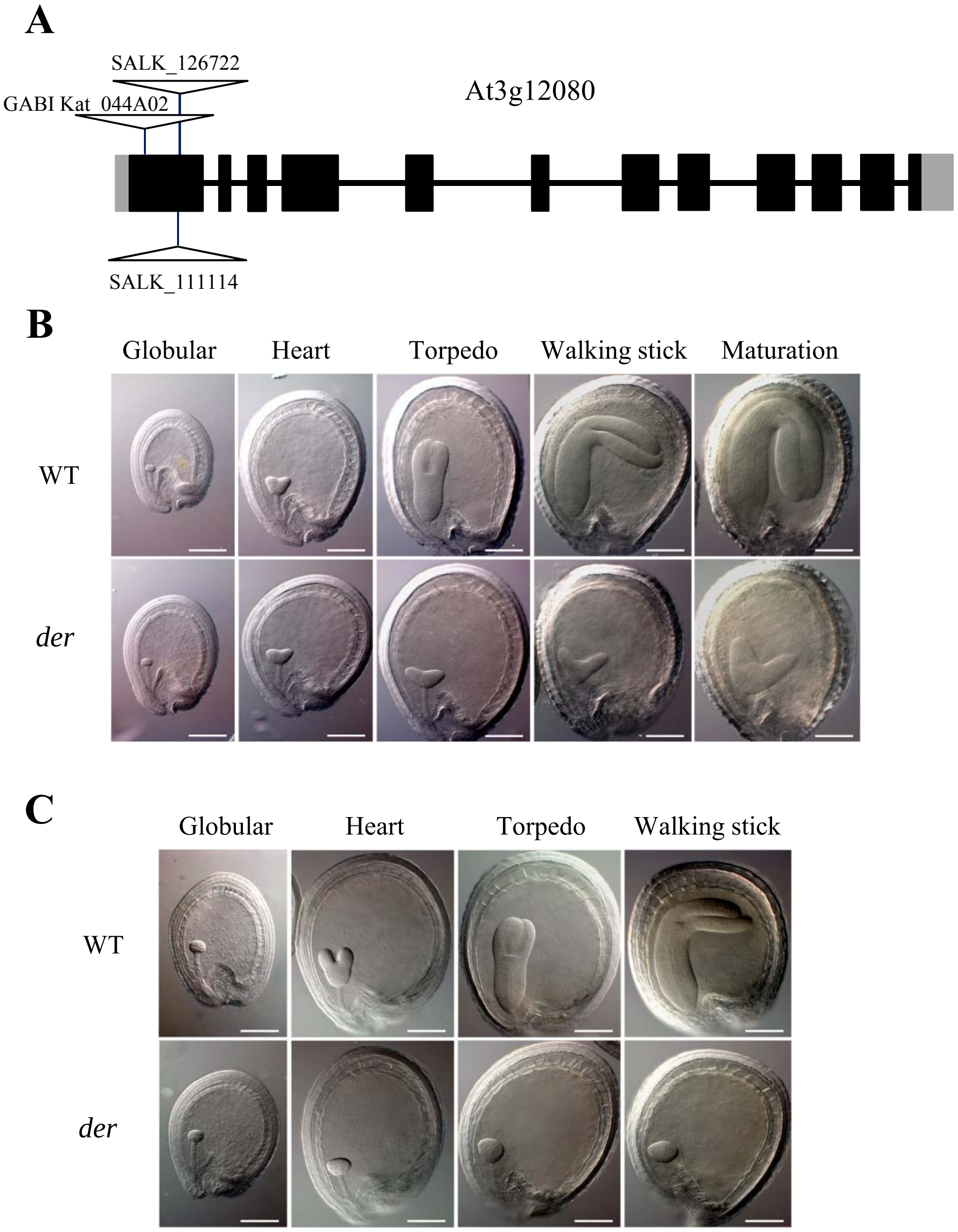

Supplementary Fig. S4. An embryonic lethal phenotype of the *Arabidopsis der* mutant.

(A) Schematic representation of T-DNA insertion sites in the genomic sequence of *Arabidopsis DER* (At3g12080). *Boxes* indicate exons, *black boxes* indicate protein-coding regions, and *gray boxes* indicate 5'- and 3'-untranslated regions. Positions of T-DNA insertion sites in three different alleles are marked.

(B, C) Representative differential interference contrast microscopy images of developing *der* embryos; growth was arrested at the heart (B) or the globular stage (C). Scale bars = 100 μm.

Supplementary Figure S5

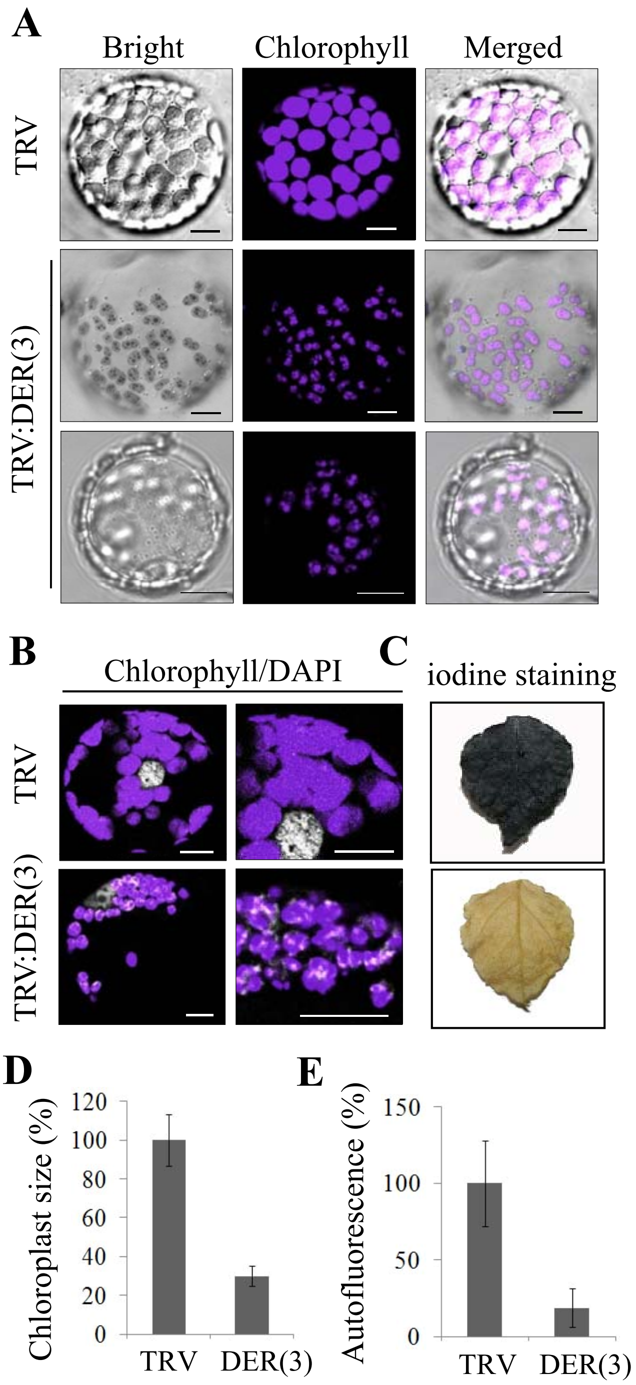

Supplementary Fig. S5. Chloroplast defects in TRV:DER VIGS lines.

(A) Leaf protoplasts from TRV and TRV:DER(3) lines were visualized by chlorophyll autofluorescence (pseudo-colored purple) using confocal laser scanning microscopy. Scale bars = 10 μm.

(B) Chloroplast nucleoids were visualized by squashing the DAPI-stained leaf protoplasts from TRV and TRV:DER(3) before observation. Chlorophyll autofluorescence (pseudo-colored purple) was visualized. Scale bars = 10 μm.

(C) Leaves from TRV and TRV:DER(3) lines were stained with iodine to detect accumulated starch (stained black) in chloroplasts.

(D) Chloroplast size was quantified by confocal microscopy. The mean diameter of TRV control chloroplasts was approximately 5.7 μm. Data points represent means ± standard deviation of 30 chloroplasts selected from 30 individual protoplasts, and were expressed as a percentage of TRV control.

(E) The average chlorophyll autofluorescence in individual leaf protoplasts was quantified by confocal microscopy. Data points represent means ± standard deviation of 30 individual protoplasts, expressed as a percentage of TRV control.

Supplementary Figure S6

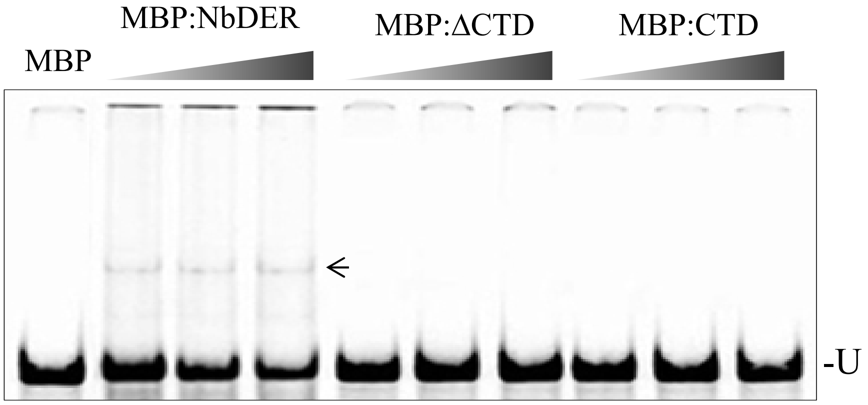

Supplementary Fig. S6. Gel-mobility shift assays of recombinant NbDER and its variants. MBP (150 pmol) and increasing concentrations (50, 100, and 150 pmol) of MBP:NbDER, MBP:ΔCTD, and MBP:CTD fusion proteins were incubated with a radiolabeled 160-nt RNA (30 ng). RNA-protein complex was resolved on a native polyacrylamide gel. Unbound (U) RNAs are indicated. The *arrow* indicates a long radiolabeled RNA generated from an uncut vector, which was occasionally observed.
