# Supplementary material for: DER containing two consecutive GTP-binding domains plays an essential role in chloroplast ribosomal RNA processing and ribosome biogenesis in higher plants
Source: J Exp Bot. 2013 Nov 23;65(1):117–30. doi: 10.1093/jxb/ert360 (PMC3883289; doi:10.1093/jxb/ert360)
Supplement: Supplementary Data [file supp_ert360_jexbot103218_file002.pdf]

Supplementary Methods S1

Materials and methods

Virus-induced gene silencing

Functional genomics using Tobacco Rattle Virus (TRV)-based virus-induced gene silencing was carried out in *N. benthamiana* as described (Cho *et al.* 2004; Kang *et al.* 2010; Jeon *et al.* 2012). Various *NbDER* cDNA fragments were polymerase chain reaction (PCR)-amplified and cloned into the pTV00 vector containing part of the TRV genome (Cho *et al.*, 2004) using *Bam*HI and *Apa*I sites. The recombinant pTV00 plasmids and pBINTRA6 vector containing RNA1, which is required for virus replication, were separately transformed into *Agrobacterium tumefaciens* GV3101. The third leaf of *N. benthamiana* (3-week-old plants) was pressure-infiltrated with the *Agrobacterium* suspension. The fourth leaf above the infiltrated leaf was used for real-time quantitative reverse transcription (RT)-PCR to detect gene silencing.

Agrobacterium-mediated transient expression

Agroinfiltration was carried out as described previously (Voinnet *et al.*, 2003). Agrobacterial cultures (GV3101) containing various constructs fused to the CaMV35S promoter were adjusted to OD_{600}=0.6 in MES buffer (10 mM MES, pH 7.5, 10 mM $MgSO_4$). The suspension was incubated with acetosyringone for 2–3 h at a final concentration of 150 µM, and infiltrated into leaves of wild-type *N. benthamiana* plants. In all experiments, *Agrobacterium* C58C1 carrying the 35S:p19 construct (Voinnet *et al.*, 2003) was co-infiltrated to achieve maximum levels of protein expression. Expressed proteins were analyzed at 48 h post-infiltration.

Real-time quantitative RT-PCR

Real-time quantitative PCR was carried out as described previously (Jeon *et al.*, 2012). The primer sets used for detection of endogenous *DER* transcripts were *DER-A* (5'-agatcattttggggaaacca-3' and 5'-tcgaaatggtaagcacacctc-3') and *DER-B* (5'-tgggaagagaggacgtgttt-3' and 5'-acaaatgtcggtggtcgaa-3').

RNA gel blot analysis

For RNA gel blot analysis, total RNA was prepared with TRIzol^{TM} Reagent (Gibco-BRL) following the manufacturer's instructions. RNA gel blot analyses were performed with approximately 20 μg total RNA as described previously (Jeon *et al.*, 2012). To generate probes, cDNAs were PCR-amplified using published sequences and cloned for sequence verification. Probes were labeled with a DecaLabel DNA Labeling Kit (Thermo Scientific). The primer sets used for PCR amplification of the probes were as follows: 16S rRNA (5'-tctcatggagagttcgatcctggctc-3' and 5'-aaaggaggtgatccagccgcaccttc-3'); 23S rRNA (5'-ttcaaacgaggaaaggcttacggtgg-3' and 5'-aggagagcactcatcttggggtggg-3'); 4.5S rRNA (5'-gaaggtcacggcgagacgagc-3' and 5'-gttcaagtctaccggtctgttagg-3'); 5S rRNA (5'-tattctggtgtcctaggcgtagagg-3' and 5'-tcctggcgtcgagctatttttcca-3'); probe 23S-1 (5'-ttcaaacgaggaaaggcttacggtgg-3' and 5'-agcacgacgcttgtattgctctcc-3'); probe 23S-2 (5'-aacgtaaggtgtccc-3' and 5'-aagccaatcccaggg-3'); probe 23S-3 (5'-ttggcacctcgatgtcggctc-3' and 5'-aggagagcactcatcttggggtggg-3'); probe 16S-1 (5'-agcccaatgtgagttt-3' and 5'-ttcatagttgcattact-3'); and probe 16S-2 (5'-caaggagagctattgc-3' and 5'-gtcgtgcgagcctccc-3'). The sizes of the probes are as follows: 1.491 kb (16S-full), 2.81 kb (23S-full), 103 bp (4.5S), 121 bp (5S), 300 bp (probe 23S-1), 600 bp (probe 23S-2), 300 bp (probe 23S-3), 229 bp (probe 16S-1), and 302 bp (probe 16S-2).

DAPI staining

DAPI staining and detection by confocal laser scanning microscopy was performed as described in Cho *et al.* (2004).

Confocal microscopy for subcellular localization of NbDER

NbDER cDNAs corresponding to the full-length coding region (Met-1 to Ala-651) and the N-terminal transit peptide (Met-1 to Ser-60) were cloned into the sGFP plasmid (Cho *et al.*, 2004) to generate NbDER:GFP and TP:GFP fusion proteins, respectively. The GFP fusion constructs, alone or in combination with STF:RFP (Jeon *et al.*, 2012) and SiR:RFP constructs (Kang *et al.*, 2010), were introduced into protoplasts isolated from *N. benthamiana* seedlings as described previously (Cho *et al.*, 2004). After 24 h, expression of the GFP and RFP fusion constructs was monitored by confocal laser scanning microscopy (Carl Zeiss LSM 510).

Transmission electron microscopy

Cotyledons and leaves were fixed with 2.5% (v/v) glutaraldehyde and with 1% osmium

tetraoxide, followed by dehydration through an ethanol series, and embedded in Spurr's resin (EM Sciences, USA). Thin sections were prepared with a LKB III ultramicrotome and stained sequentially with 5% uranyl acetate and 3% lead citrate, and observed under a JEOL 1200 EXII transmission electron microscope.

Immunoblotting

Membrane preparation and Western blotting were performed according to the manufacturer's instructions using polyclonal rabbit antibodies against rbcL, cyt f, D1, and atpB (1:5,000, 1:3,000, 1:10,000, and 1:5,000 dilution, respectively; Agrisera), and horseradish peroxidase-conjugated goat anti-rabbit IgG antibodies (1:5,000 dilution; GE Healthcare). Signals were detected using an electrochemiluminescence kit (Amersham Pharmacia) on Kodak X-ray films. Signal intensities were quantified using the Analysis Life Science Research imaging system (Olympus).

Chloroplast fractionation and NbDER localization

The NbDER:GFP construct was agroinfiltrated into leaves of *N. benthamiana* plants. After 48 h, chloroplast stroma and thylakoid membrane fractions were prepared from the infiltrated leaves as described previously (Kwon and Cho, 2008). The supernatant and pellet fractions were analyzed by sodium dodecyl sulfate polyacrylamide gel electrophoresis (SDS-PAGE) and immunoblotting using the polyclonal antibodies against GFP (Clontech; 1:3,000), rbcL (Agrisera; 1:5,000), and D1 (Agrisera; 1:10,000) as described previously (Jeon *et al.*, 2012).

Purification of recombinant proteins

To purify recombinant proteins of NbDER and its mutants for GTPase assays, the corresponding *NbDER* cDNA fragments were PCR-amplified and cloned into the pMAL^{TM}c2 vector (New England Biolabs). The MBP fusion proteins were purified using amylose resin following the manufacturer's instructions (New England Biolabs). Purified proteins of MBP:NbDER, MBP:PM1, MBP:PM2, and MBP:PM1/2 were concentrated using Amicon Ultra Centrifugal Filters (Millipore). To purify MBP:CTD for RNA-binding assays, the *NbDER* cDNA fragment corresponding to amino acid residues 520–651 was PCR-amplified and cloned into the pMAL^{TM}c2 vector (New England Biolabs).

RNA binding assay

To prepare 16S and 23S rRNA, the cDNAs encoding full-length 16S and 23S rRNA were cloned into the pGEM T-easy vector. The constructs were digested with *BamH*I restriction enzyme, and RNAs were prepared by *in vitro* transcription using T7 RNA polymerase (Promega). For RNA binding assays, the RNA substrates were incubated with the purified recombinant MBP fusion proteins in binding buffer (10 mM Tris-HCl, pH 7.5, 50 mM NaCl, 1 mM EDTA, 7.4% glycerol) on ice for 30 min. The reaction mixtures were loaded on 0.8% agarose gel, and RNA bands were visualized by UV light after ethidium bromide staining or by PhosphorImager (GE Healthcare Life Sciences). For EMSA competition assays, 25 picomoles of the recombinant proteins were incubated with variable ratios of radiolabeled (200 ng) and unlabeled RNAs, ranging from 1:0 to 1:20. A sequence-nonspecific ^{33}P-labeled RNA substrate (~160 nucleotides in length) was prepared by transcribing *BamH*I-digested pET-22b(+) plasmid using T7 RNA polymerase as described previously (Jeon *et al.*, 2012). For RNA-protein interaction analysis, the labeled sequence-nonspecific RNAs (30 ng) were incubated with purified recombinant proteins in binding buffer (10 mM Tris-HCl, pH 8.0, 50 mM NaCl, 1 mM EDTA, and 7% glycerol) for 30 min on ice. The mixture was separated on 6% non-denaturing polyacrylamide gel, and RNA bands were detected by PhosphorImager.

GTPase assay

The turnover rate (k_{cat}) of recombinant proteins of NbDER and its mutants was measured as described previously (Im *et al.*, 2011). A reaction mixture containing 3 μM recombinant proteins and 1 mM GTP in GTPase assay buffer (20 mM Hepes, pH 8, 1 mM $MgCl_4$, 0.5 mM DTT, and 1 mM NaN_3) was incubated at room temperature for 18 h. The released phosphate was quantified using the Biomol green reagent (Biomol Research Laboratories) according to the manufacturer's protocol. The catalytic constant was derived from the equation $k_{cat} = V_{max}/C_{recombinant\ protein}$.

Sucrose density gradient analysis

GFP-fusion proteins of NbDER and its variants were expressed in *N. benthamiana* leaves by agroinfiltration, and the leaf extracts were fractionated through 15%–55% sucrose density gradients as described previously (Barkan 1993; Williams and Barkan, 2003). Proteins extracted from the fractions were separated by SDS-PAGE and subjected to immunoblotting with anti-GFP antibodies (Clontech) and anti-RPL10 antibodies (Santa Cruz Biotechnology).

For polysome analysis, leaf extracts of TRV and TRV:NbDER VIGS plants were fractionated through 15%–55% sucrose density gradients. Total RNA was extracted from sucrose density gradient fractions and subjected to RNA gel blot analysis.

Supplementary References

Barkan A 1993. Nuclear mutants of maize with defects in chloroplast polysome assembly have altered chloroplast RNA metabolism. *The Plant Cell* 5, 389-402.

Im CH, Hwang SM, Son YS, Heo JB, Bang WY, Suwastika IN, Shiina T, Bahk JD. 2011. Nuclear/nucleolar GTPase 2 proteins as a subfamily of YlqF/YawG GTPases function in pre-60S ribosomal subunit maturation of mono- and dicotyledonous plants. *Journal of Biological Chemistry* **286,** 8620-8632.

Kwon KC, Cho MH. 2008. Deletion of the chloroplast-localized AtTerC gene product in *Arabidopsis thaliana* leads to loss of the thylakoid membrane and to seedling lethality. *The Plant Journal* **55,** 428-442.

Voinnet, O., Rivas, S., Mestre, P. and Baulcombe, D. 2003. An enhanced transient expression system in plants based on suppression of gene silencing by the p19 protein of tomato bushy stunt virus. *The Plant Journal* **33,** 949–956.

Williams PM, Barkan A. 2003. A chloroplast-localized PPR protein required for plastid ribosome accumulation. *The Plant Journal* **36,** 675-686.
